# Supplementary material for: Network fixation expansion microscopy
Source: bioRxiv. 2026 Oct 9:2026.10.07.756227. Preprint. [Version 1] doi: 10.64898/2026.10.07.756227 (PMC13692233; doi:10.64898/2026.10.07.756227)
Supplement: Supplementary file 1 [file media-1.pdf]

Supplementary Information

Network fixation expansion microscopy

Table of Contents

Supplementary Notes 2

Supplementary Note 1: Acrolein Handling and Quality 2

Supplementary Note 2: Sub-pixel gap detection limits..................................... 2

Supplementary Note 3: Performing fixation and gelation at −20 °C.................. 3

Supplementary Figures... 4

Supplementary Figure 1. Cold processing steps alone (PFA at -20 °C) are insufficient for microtubule preservation, even with high gel monomer concentration.. 4

Supplementary Figure 2. Proposed schematic model for how fixation strategy determines microtubule structural outcomes in ExM. 5

Supplementary Figure 3. Schematic Illustration of gelation chamber assembly and UV-initiated polymerization setup for nfExM. 6

Supplementary Figure 4. How sub-pixel gaps appear in pixel-by-pixel detection. 8

Supplementary Tables 9

Supplementary Table 1. List of proteases tested with post-expansion staining in samples fixed with 3% w/v PFA and 0.1% w/v glutaraldehyde. 9

Supplementary Table 2. Cleavable fixatives tested, and their outcomes........ 20

Supplementary Table 3: Detailed Statistics for Figure 2 48

Supplementary Table 4: Detailed Statistics for Figure 3 48

Supplementary Table 5: Detailed Statistics for Figure 4 49

Supplementary Table 6: Detailed Statistics for Figure 5 49

References... 51

Supplementary Notes

Supplementary Note 1: Acrolein Handling and Quality

The acrolein-methanol solution (Restek, Cat. No. 30645) has a manufacturer-stated shelf life of 3 months. We had experience where solutions used one month past the expiration date produced poor microtubule preservation quality, similar to that observed with PFA fixation (Fig. 3a). However, we did not systematically characterize how preservation quality degrades as a function of time relative to the expiration date. We therefore recommend to always use fresh solutions that are well within their shelf life.

The manufacturer provides the acrolein-methanol solution in a glass ampule containing 1 mL of solution. This relatively small volume, even if pre-chilled to −20 °C, can be rapidly warmed well above −20 °C simply by holding the ampule in one’s hand. Therefore, prior to each experiment, we transferred the required volume of acrolein-methanol solution from the original glass ampule into an amber microcentrifuge tube in the fume hood, and then chilled the tube to −20°C in a metal cold block before use. When retrieving the solution for use, the amber tube was kept fully seated in the cold block at all times, with only the tube cap being opened (but not lifting the tube from the block) to withdraw the liquid. After adding the acrolein solution to the 24-well plate, we sealed the well with laboratory tape or adhesive plate sealing film to prevent acrolein evaporation during incubation.

Supplementary Note 2: Sub-pixel gap detection limits

Our algorithm works as follows: for each pixel, we take the average intensity of a 7-pixel window (the pixel itself plus 3 on each side), and mark it as a gap if the pixel is at least 20% dimmer than that average (i.e., its intensity < 0.8 × local mean).

The smallest gap we can report is 1 pixel (0.041 µm). What happens to a gap smaller than 1 pixel depends on where it lands on the pixel grid — see **Supp. Figure 4** for three scenarios (using a 0.3-pixel-wide gap as an example for scenarios B and C). For illustration we assume a uniform intensity of 100 along an intact microtubule; real data has fluctuations from noise and staining variation, but the alignment-dependent behavior we describe holds regardless.

- **Scenario A**: A gap that fills roughly one whole pixel drops that pixel's intensity close to zero, which easily crosses the threshold. Reported as a 1-pixel gap.
- **Scenario B**: A sub-pixel gap (e.g., 0.3 pixel wide) that sits inside one pixel still drops that pixel's intensity enough (e.g., from 100 to 70) to cross the threshold. Also reported as a 1-pixel gap — even though the real gap is smaller.
- **Scenario C**: The same 0.3 pixel wide gap that spanning two pixels splits the intensity drop between them. Neither pixel drops enough to cross the threshold, so the gap is missed entirely (reported as 0).

So a 1-pixel gap in our data could be a real 1-pixel gap (scenario A), or a sub-pixel gap that happened to land inside one pixel (scenario B). Gaps that crossed pixel boundaries (scenario C) don't show up at all, if too small.

Supplementary Note 3: Performing fixation and gelation at −20 °C

To ensure the sample and reagents were maintained at −20 °C for the duration of the nfExM fixation, monomer incubation, and gelation steps (see Methods section, “Fixation, gelation, and denaturation of cell culture with nfExM,” for details of the nfExM treatments), we typically did the following:

- Beginning just after the addition of −20 °C acrolein-methanol solution, the 24-well plate containing coverslip with cells was placed and kept on a pre-chilled thermal workstation with a metal surface (Corning CoolBox XT 432021 with Corning XT Freezing Core 432082 and Corning CoolSink XT96F 432070) to hold the plate and sample at −20 °C as it was moved between steps.
- To perform the −20 °C incubations (in acrolein-methanol or monomer solution), the entire well plate-thermal workstation assembly was moved into a −20 °C freezer.
- Before adding monomer solution and coverslip with cells, the gelation chamber slide was pre-chilled and maintained at −20 °C by placing it on a separate metal cold block pre-chilled to –20 °C.
- The 27 µL drop of monomer solution was placed on this chilled gelation chamber immediately (5–20 s) prior to closing the chamber with the coverslip containing cells.
- The closed gelation chamber along with the metal cold block was immediately (within 60 s) transferred to a −20 °C freezer.
- The UV exposure was then performed by placing the UV exposure unit (**Supp. Fig. 3**) directly above the gelation chamber inside of the −20 °C freezer.

Alternatively, this entire procedure can be carried out in a −20 °C cold room if one is available.

Supplementary Figures

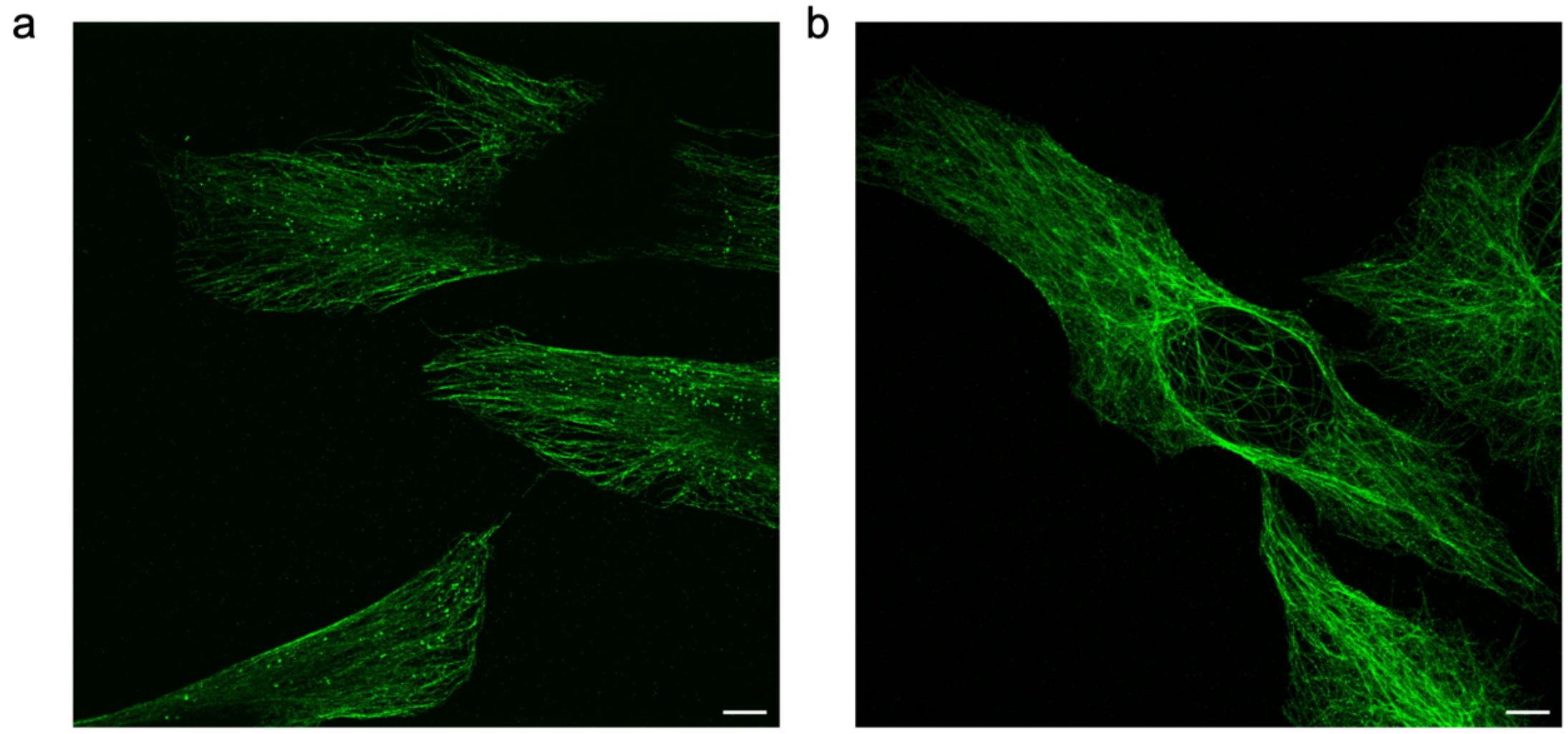

Supplementary Figure 1. Cold processing steps alone (PFA at -20 °C) are insufficient for microtubule preservation, even with high gel monomer concentration.

To test whether nfExM's improvement in protein retention comes from performing steps at low temperature rather than from its acrolein chemistry, we added cold PFA fixation steps to the proExM protocol (which uses Acryloyl-X, SE (AcX) anchoring), without introducing acrolein chemistry. These were initial tests using a single culture batch; observations are thus preliminary, but helped guide us along the path.
(a) Representative (n = 6 images from 1 culture batch) confocal image (maximum intensity z-projection) of expanded HeLa cells, post-expansion immunostained for β-tubulin. Protocol: 4% w/v paraformaldehyde (PFA) in methanol at -20°C for 10 min, followed by AcX anchoring at room temperature for 2 hrs, followed by gelation of a standard proExM gel (low total monomer concentration, or low T%, where T% denotes the total monomer percentage in units of w/v, in this case 11.1% w/v) at 37°C for 1.5 h.
(b) Representative (n = 9 images from 1 culture batch) confocal image, as in (a), but with fixation with 4% w/v PFA in methanol at -20°C for 10 min, followed by AcX anchoring at room temperature for 2 hrs, followed by gelation of an nfExM high T% (59.2% w/v) monomer solution at -20°C via UV initiation for 3 min.
All images were acquired under the same microscope settings with a 40x 1.15 NA water objective (Nikon). Images were visualized with the auto-scaling function in Fiji. Scale bar (in biological units, e.g. real space units divided by expansion factor): 5 μm.

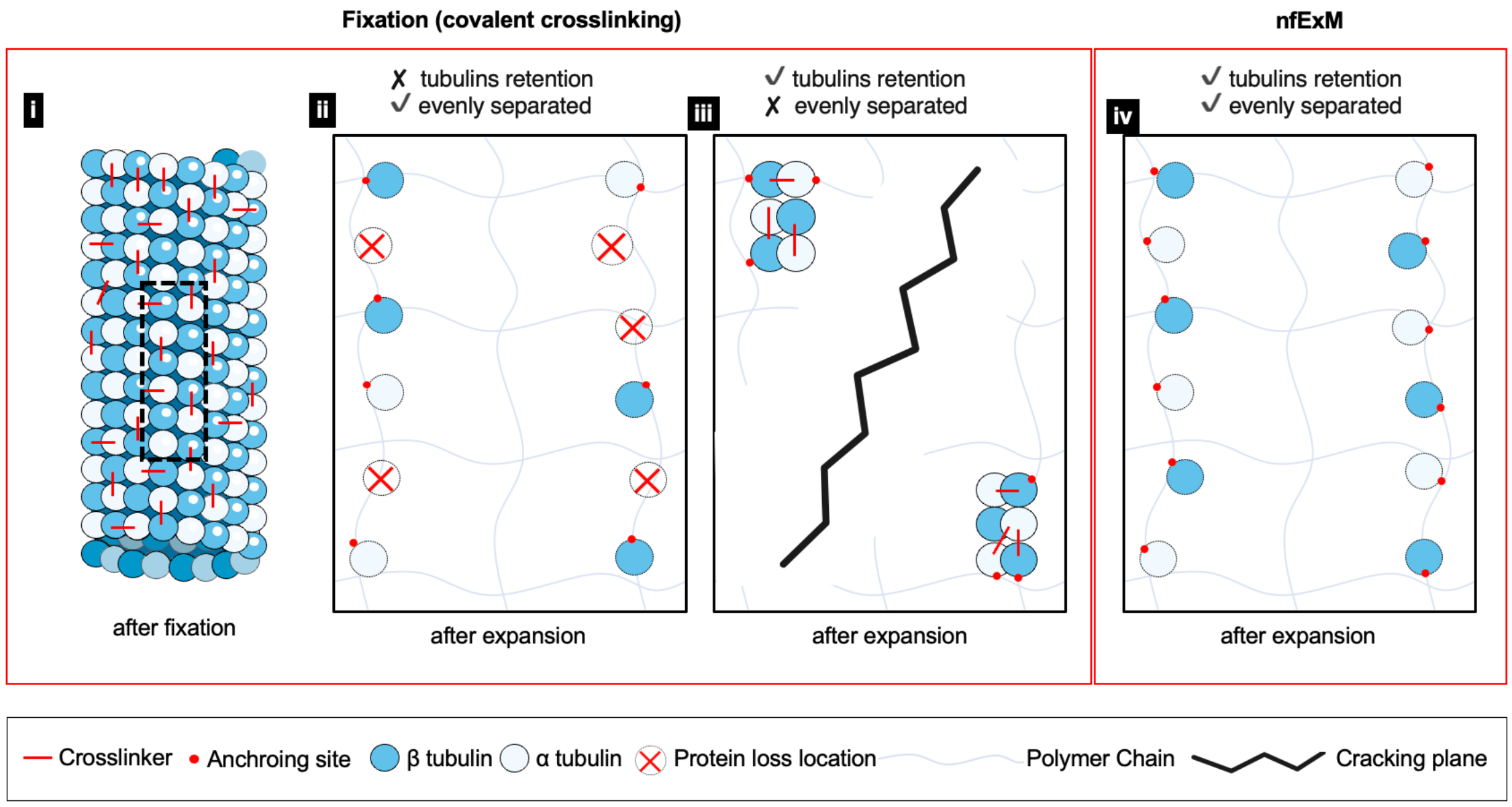

Supplementary Figure 2. Proposed schematic model for how fixation strategy determines microtubule structural outcomes in ExM.

This schematic proposes that the strength of fixation-induced protein-protein crosslinks determines how microtubule organization is preserved after expansion. Our proposed model can be used to interpret the results of three scenarios: weak fixation (panels i and ii, e.g., PFA), strong fixation (panels i and iii, e.g., GA), and protein-network fixation (panel iv, nfExM).

i) Schematic of microtubule organization after standard covalent crosslinking. Tubulin heterodimers (blue circles, β-tubulin; white circles, α-tubulin) are crosslinked (red lines) to neighboring subunits to preserve nanoscale organization. The strength of these protein–protein crosslinks determines subsequent expansion outcomes. Black dashed line box shows the region of interest highlighted in panels ii, iii, and iv.

(ii) Weak fixation scenario (e.g., PFA): expansion with weak protein–protein crosslinking leads to the loss of many tubulin subunits (red X, protein loss locations), although the gel expands uniformly (✓ evenly separated). This is consistent with the reduced intensity and increased gap size and ratio observed in standard proExM and CryoExM (Fig. 2d–f).

(iii) Strong fixation scenario (e.g., GA): expansion with extensive protein–protein crosslinking. Glutaraldehyde creates rigid protein assemblies (tubulins at the top left and bottom right) that resist protein loss (✓ tubulin retention) but impede uniform gel swelling. Mechanical stress from these rigid assemblies could generate visible cracks (zigzag, cracking plane) and non-uniform expansion (✗ evenly separated; deformed polymer chains) during expansion. This is consistent with good microtubule continuity (Fig 4o-p) but frequent cracking (Fig 4t) in GA-based methods.

(iv) nfExM strategy: Tubulin subunits are anchored directly to the polymer network. During expansion, proteins move with the gel, achieving both high retention (✓ tubulin

retention) and uniform separation (✓ evenly separated). This is consistent with nfExM's minimal gaps (Fig 4o–p), yet zero cracking (Fig 4t).
This schematic offers a hypothesis for how fixation strategy shapes ExM outcomes: weak fixation preserves gel uniformity but loses protein; strong conventional crosslinking preserves protein but causes cracking; our nfExM strategy (anchoring proteins directly to the gel) retains both protein and gel uniformity. The model is consistent with our observed intensity, gap, and cracking measurements (Figs. 2, 3, 4), but direct biophysical validation (e.g., gel mechanics, protein-gel binding forces) would be needed to test these mechanisms.

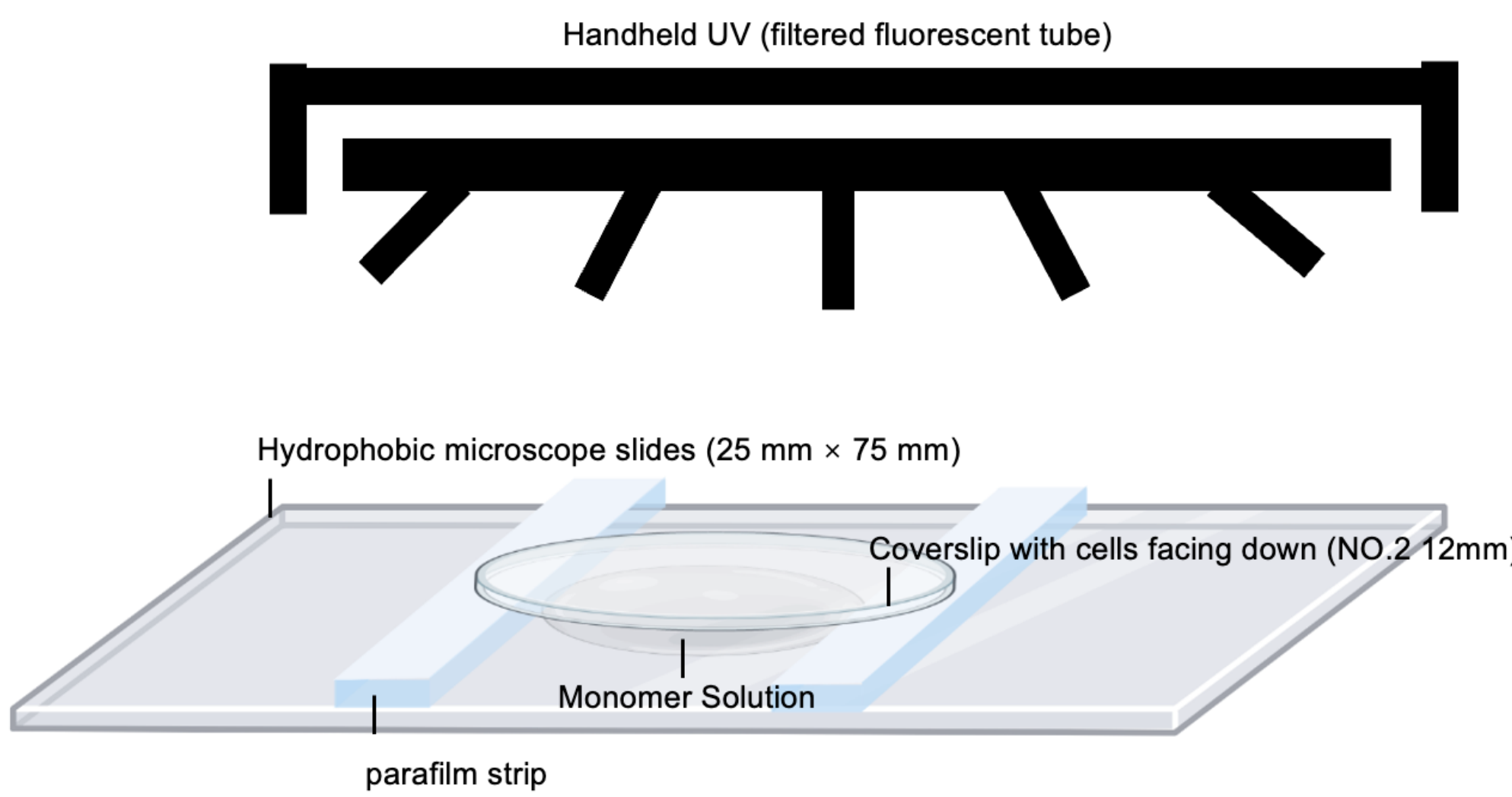

Supplementary Figure 3. Schematic Illustration of gelation chamber assembly and UV-initiated polymerization setup for nfExM.

The schematic illustration depicts the components of the custom-built gelation chamber used for nfExM. The chamber consists of hydrophobic microscope slides (25 mm × 75 mm) serving as the base, which provides a non-adherent surface for the polymerized gel to facilitate peeling the polymerized gel from the slide in a later step; see Methods section, "Gelation Chamber Preparation" for the silane-based hydrophobic treatment protocol. Note: as a simpler alternative, wrapping the slide surface with a layer of Parafilm prior to chamber assembly also provides a sufficient non-adherent surface for gel release without chemical treatment. A monomer solution is placed at the center of two spacer strips (~ 27 µL for cells cultured on 12 mm diameter coverslips), and a No. 2 coverslip containing the cells is positioned cell-side down on top of the monomer solution to close the chamber. To control the gel thickness, the coverslip is supported by two

parallel Parafilm strips (or a No.2 coverslip) placed ~10 mm apart (for a 12 mm diameter coverslip). This spacing defines the final thickness of the polymerized gel. The chamber is then exposed to UV light emitted from a handheld UV lamp (Analytik Jena, catalog number #95-0343-01) with a center wavelength of 365 nm, positioned approximately 4 cm from the sample. The UV power density at the sample was measured at 0.0171 mW/mm^2 using a power meter (Thorlabs), with an exposure duration of 3 minutes. The entire assembly is maintained at -20°C until the polymerization process is complete, typically by working inside a -20°C freezer or a -20°C cold room.

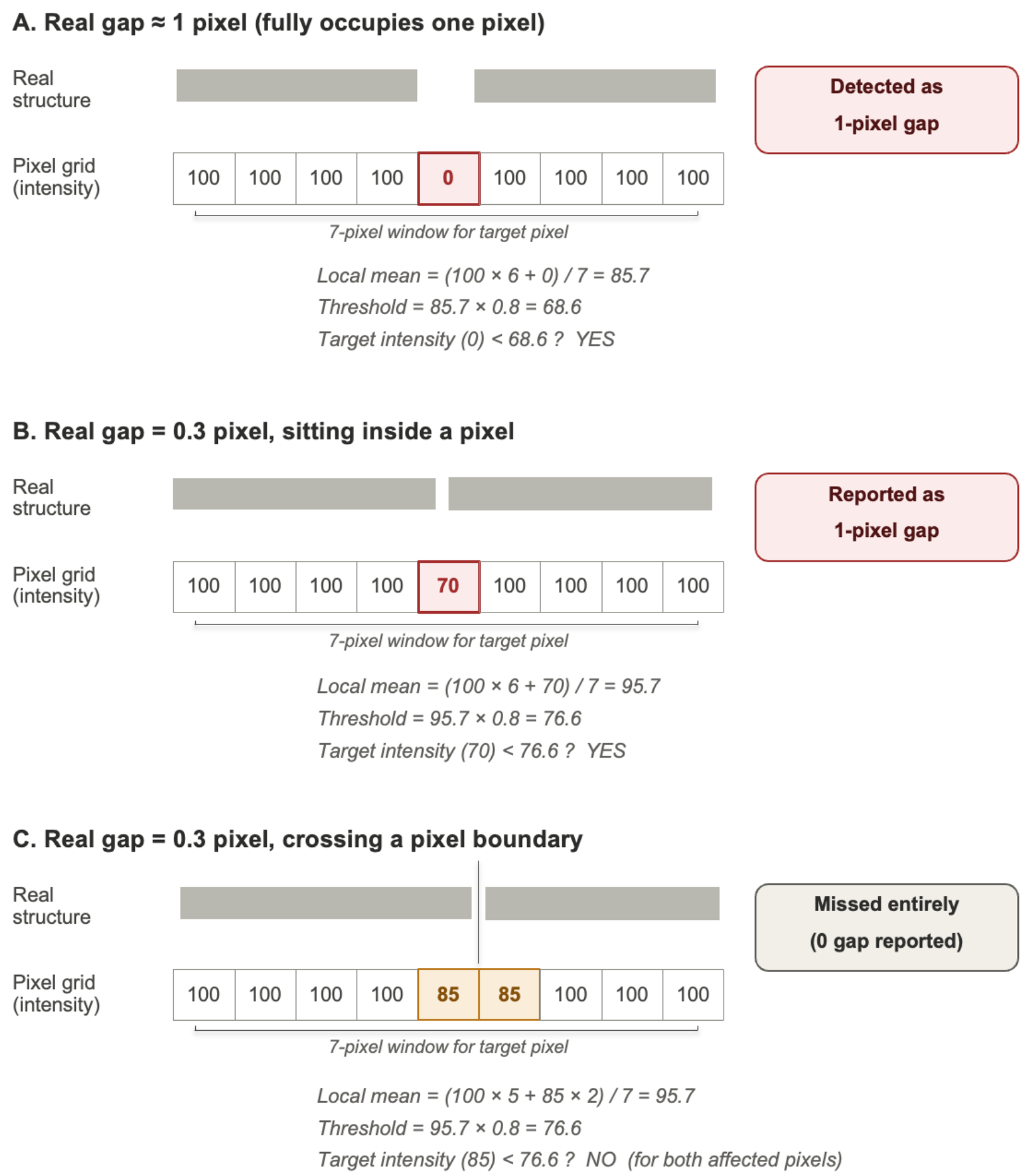

Supplementary Figure 4. How sub-pixel gaps appear in pixel-by-pixel detection.

Three scenarios showing why a gap smaller than 1 pixel can either show up as a 1-pixel gap or be missed entirely, depending on how it aligns with the pixel grid. Scenarios B and C use a 0.3-pixel-wide gap as an example. For illustration, gap-free microtubule regions are shown with a uniform intensity of 100; real data has noise, but the alignment-dependent behavior shown here does not depend on this simplification. Gray bars: the real microtubule structure. Squares below: the pixel grid our imaging samples, with the intensity value for each pixel. Target pixels (the ones being tested for gap) are highlighted — red when they cross the threshold (detected), amber when they don't (missed). Calculations below each scenario show local mean, threshold (local mean × 0.8), and whether the target pixel's intensity is below threshold.

Supplementary Tables

Supplementary Table 1. List of proteases tested with post-expansion staining in samples fixed with 3% w/v PFA and 0.1% w/v glutaraldehyde.

HeLa cells were cultured on 12 mm glass coverslips in 24-well plates. Cells were fixed with freshly prepared 3% w/v PFA, 0.1% w/v GA in PBS for 10 min at room temperature (RT), followed by reduction with 0.1% w/v sodium borohydride in 1× PBS for 7 min at RT and then quenching with 100 mM glycine in 1× PBS for 10 min at RT. After fixation, cells were washed three times with 1× PBS for 5 min each at RT. To equip proteins with polymerizable handles, cells were then incubated with 0.1 mg/mL Acryloyl-X, SE (AcX) in PBS (diluted from a 10 mg/mL stock in anhydrous DMSO) for 2h at RT without shaking.

Prior to gelation, a gelation chamber was constructed on a standard glass slide (25 × 75 mm). Spacers were created by cutting strips (~0.3 cm wide) from the standard 4-inch (~10 cm) width of a Parafilm M roll. Each strip was wrapped twice around the slide to form a spacer of defined thickness (~260 μm). The spacers were positioned ~10 mm apart to accommodate the 12 mm coverslip (see **Supp. Fig. 3** for chamber construction).

Gelation was performed using a proExM monomer solution consisting of 8.625% w/v sodium acrylate, 2.5% w/v acrylamide, 0.15% w/v N,N′-methylenebisacrylamide, and 2 M NaCl in 1× PBS. Polymerization was initiated by adding ice-cold stock solution of 10% w/v TEMED and 10% w/v APS to the ice-cold proExM monomer solution to a final concentration of 0.2% w/v TEMED and 0.2% w/v APS, with APS added last. The solution was added to the 24-well plate containing coverslips, and then incubated on ice for 3 min to permeate the specimen with monomers. The coverslip was then removed from the well and placed cell-side down onto ~27 μL of ice-cold monomer solution (containing TEMED and APS) deposited at the center of the gelation chamber at RT. Additional solution was added from the side to fill the chamber if necessary, and the coverslip was gently pressed to ensure contact with the spacers, yielding a uniform gel thickness. Finally, the assembly was placed in a humidified box and incubated at 37°C for 1.5 h for gelation.

Following gelation, the gel was removed from the chamber, trimmed with a razor blade into an approximately 5 mm × 5 mm square shape, then one corner was diagonally removed to create an asymmetric fiducial marker for orienting the cell-containing region during imaging. The trimmed gel was transferred to a well of a 24-well plate containing the specific enzyme digestion buffer (buffer only, enzyme-free) for equilibration, 30 minutes at RT. The buffer was then replaced with fresh buffer containing the enzyme, and the gel was incubated under protease-specific conditions (see table for buffer compositions, enzyme concentrations, temperatures, and incubation times). Following digestion, the gel was washed with PBS for 15 min followed by three washes of 30 min each. The gel was then subjected to immunostaining (at which point gels were partially expanded to ~ 1.5-2x). First, the gel was blocked with MAXblock blocking medium

(Active Motif, 15252) for 30–60 minutes at RT, followed by a 10 min wash with washing medium (MAXwash, Active Motif, 15254) at RT. The gel was then incubated with rabbit anti-beta-tubulin primary antibody (abcam, ab6046, 1:100 dilution) in staining medium (MAXbind, Active Motif, 15253) for 5 hours at RT or 16h at 4°C. After three 30-minute washes with PBS, the gel was incubated with goat anti-rabbit Alexa Fluor 546 secondary antibody (Thermo Fisher, A-11035; 1:100 dilution) staining medium for 5 hours at RT or 16h at 4°C, followed by one wash with PBS for 30 min. Finally, the gel was expanded in deionized water for two 30-minute sessions, followed by a 16h incubation in deionized water at RT to allow for full expansion. Expanded gels were imaged promptly thereafter as prolonged storage in pure water may reduce post-expansion antibody signal due to antibody dissociation in low-ionic-strength conditions[1].

To evaluate gel integrity, phenotypes were defined as follows: “Gel cracking” refers to >1 µm-scale (physical units) fractures of the hydrogel observed in cell-containing samples, where the gel exhibits visible fractures that cut across the cell and abruptly truncate or displace microtubule bundles at the crack planes. “Microtubule discontinuity” refers to fragmentation of the microtubule labeling into short segments or puncta with frequent, e.g. ~150-500 nm (physical units; the lower limit is based on what is detectable using the pixel gap method, see **Fig. 2i, 3h, 4s**, and **Supp. Fig. 4)** gaps along the filaments, in contrast to continuous microtubules that can be traced for long distances.

All image scale bars in the table are 50 µm (in physical units).

Protocol references are A: https://www.neb.com/en-us/protocols/2016/09/14/in-gel-digestion-protocol-for-endoproteinase-lysc-p8109 ; B: https://www.promega.com/resources/protocols/product-information-sheets/n/gluc-sequencing-grade-protocol/ ; C: https://www.promega.com/products/mass-spectrometry/proteases-and-surfactants/rlys-c-mass-spec-grade/ ; D: https://www.promega.com/products/mass-spectrometry/proteases-and-surfactants/arg_c_-sequencing-grade/ ; E: https://www.promega.com/products/mass-spectrometry/trypsin/trypsin-lys-c-mix-mass-spec-grade?catNum=V5071#protocols ; F: https://www.promega.com/products/mass-spectrometry/proteases-and-surfactants/chymotrypsin_-sequencing-grade/ .

Enzyme(s)	Vendor	Cat#	Condition	Results
Trypsin	New England Biolabs	P8101S	50 ug/mL<br>50 mM Tris, pH 8<br>5 mM $CaCl_2$<br>37℃, 16h<br>Modified from ref. [2]	Microtubules appeared discontinuous<br>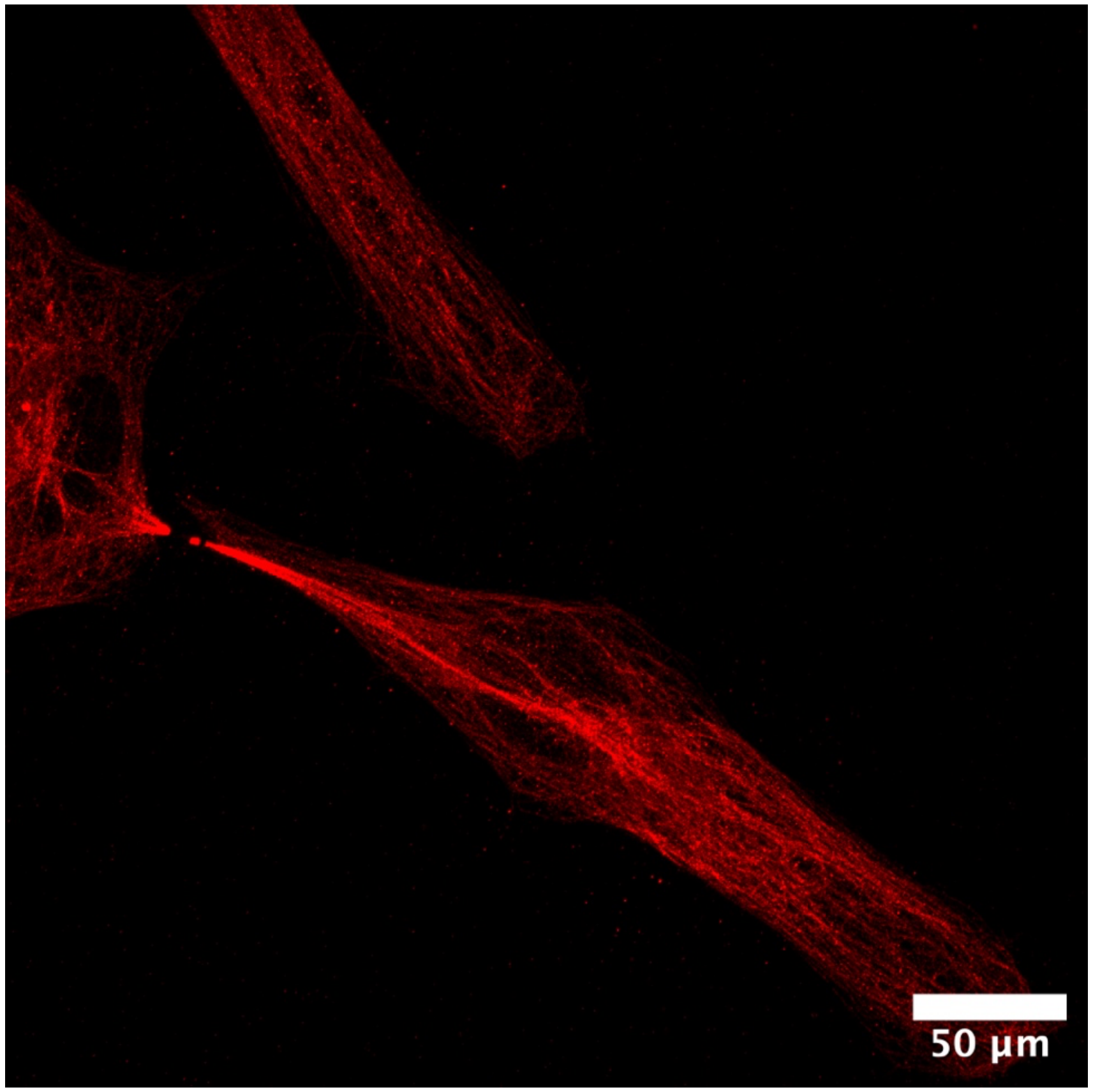<br><br>Brightness and contrast settings: first set by the ImageJ auto-scaling function and then manually adjusted to improve contrast for the stained structures of interest (done throughout the images in Supp. Table 1)

LysC	New England Biolabs	P8109S	20 ug/mL<br>50 mM Tris, pH 8.5<br>1 mM EDTA<br>37℃, 16h<br>Modified from protocol reference A.	Microtubules appeared discontinuous<br>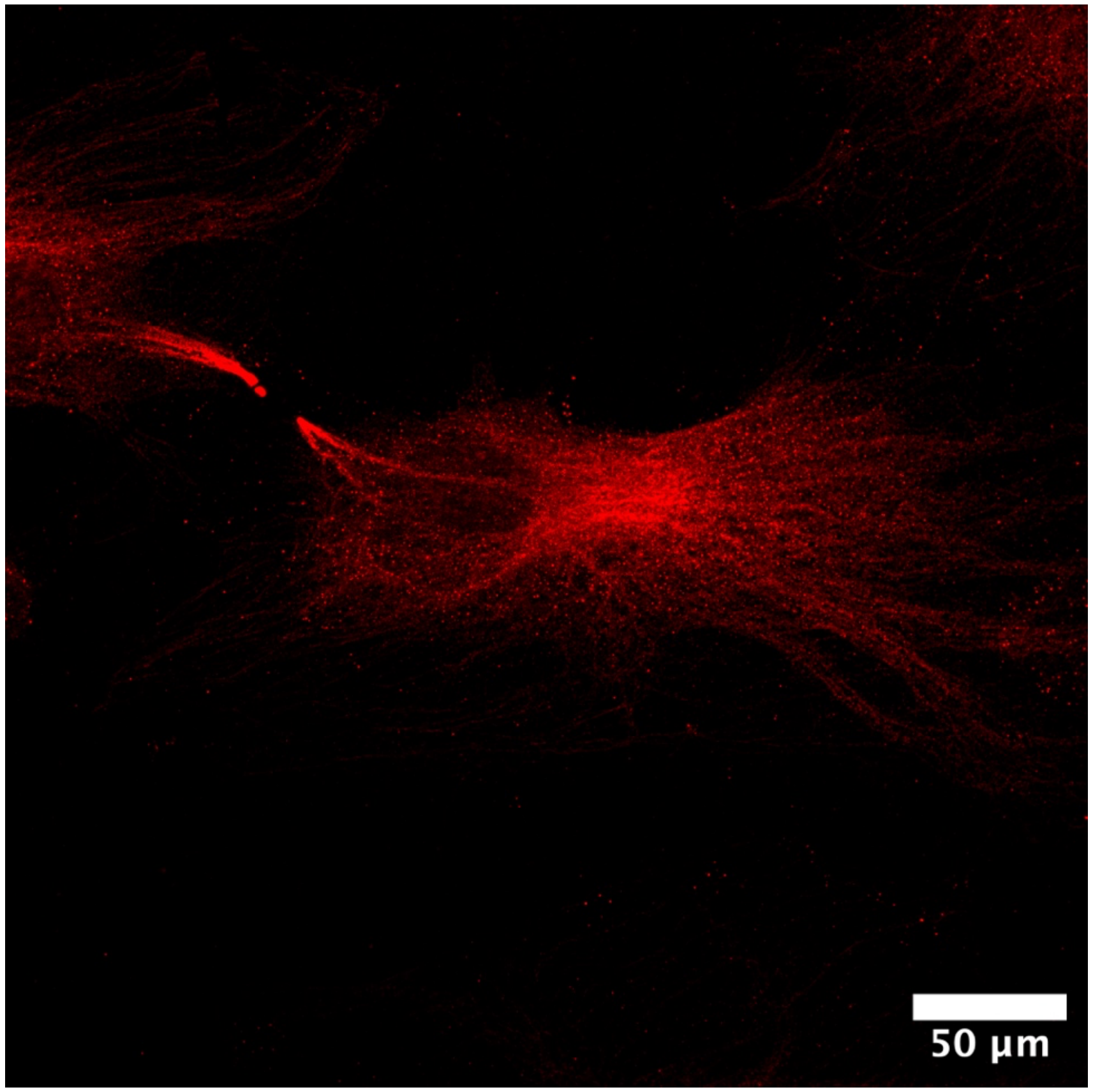
GluC	New England Biolabs	P8100S	50 ug/mL<br>100 mM ammonium bicarbonate buffer, pH 8<br>0.5 mM Glu-Glu<br>25℃, 16h	Cracks were observed within the gel & microtubules appeared discontinuous

			Modified from protocol reference B.	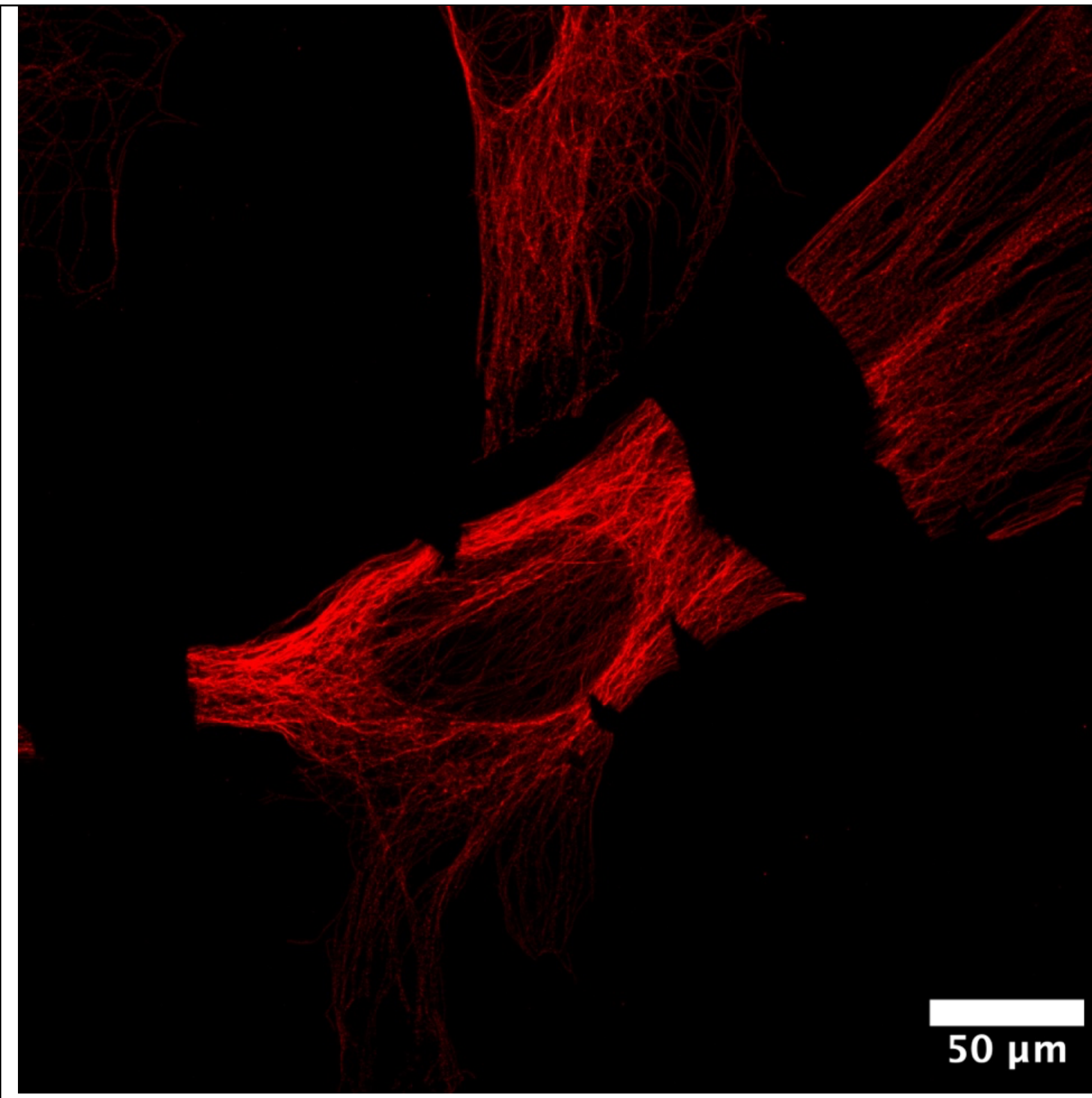
AspN	New England Biolabs	P8104S	50 ug/mL<br>50 mM ammonium bicarbonate buffer, pH 8<br>2.5mM $ZnSO_4$<br>37℃, 16h<br>Modified from protocol reference B.	Microtubules appeared discontinuous

				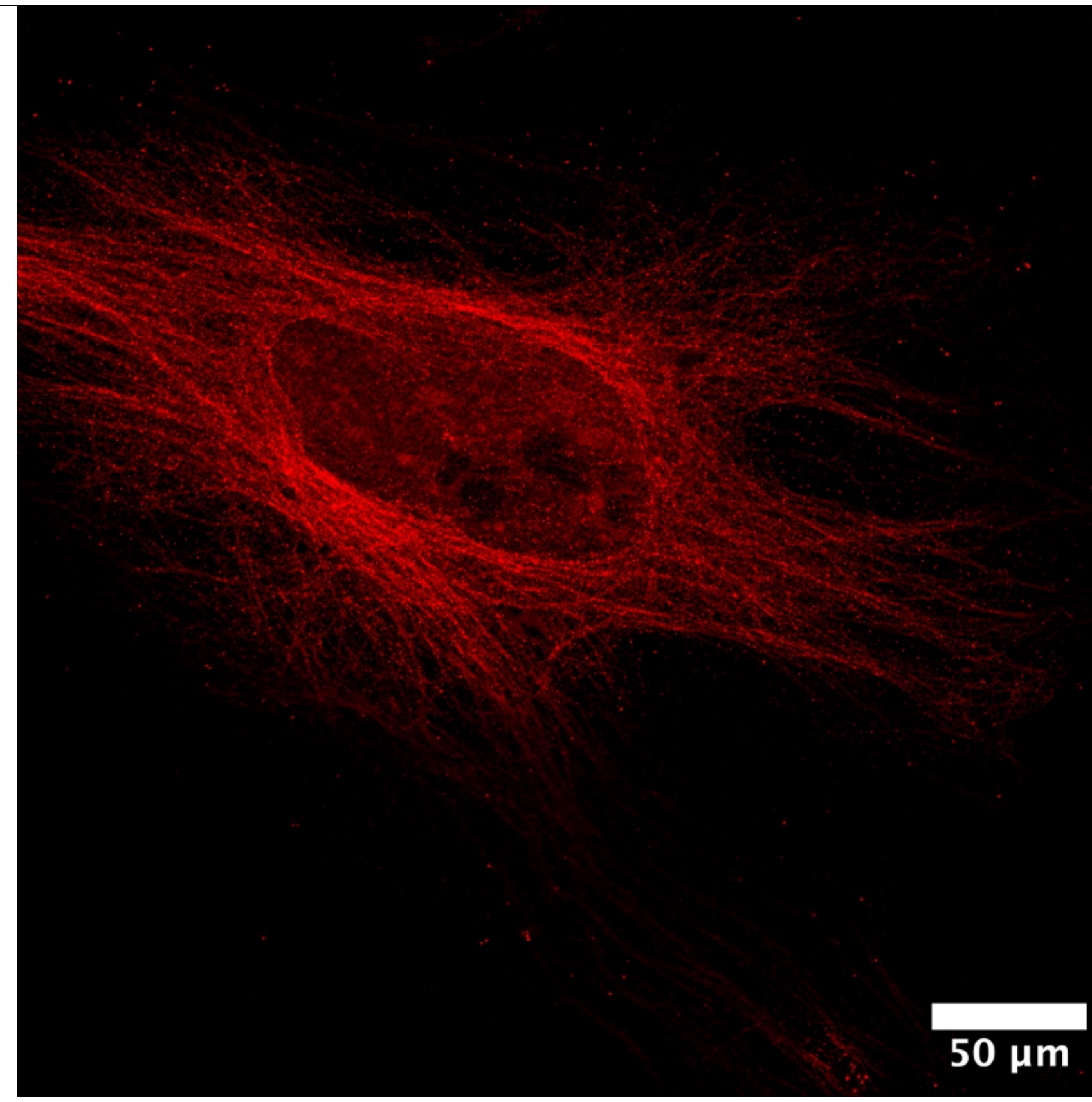
rLys-C	Promega	V1671	15 ug/mL<br>50 mM Tris, pH 8.5<br>1 mM EDTA<br>37℃, 16h<br>Modified from protocol reference C.	Microtubules appeared discontinuous

				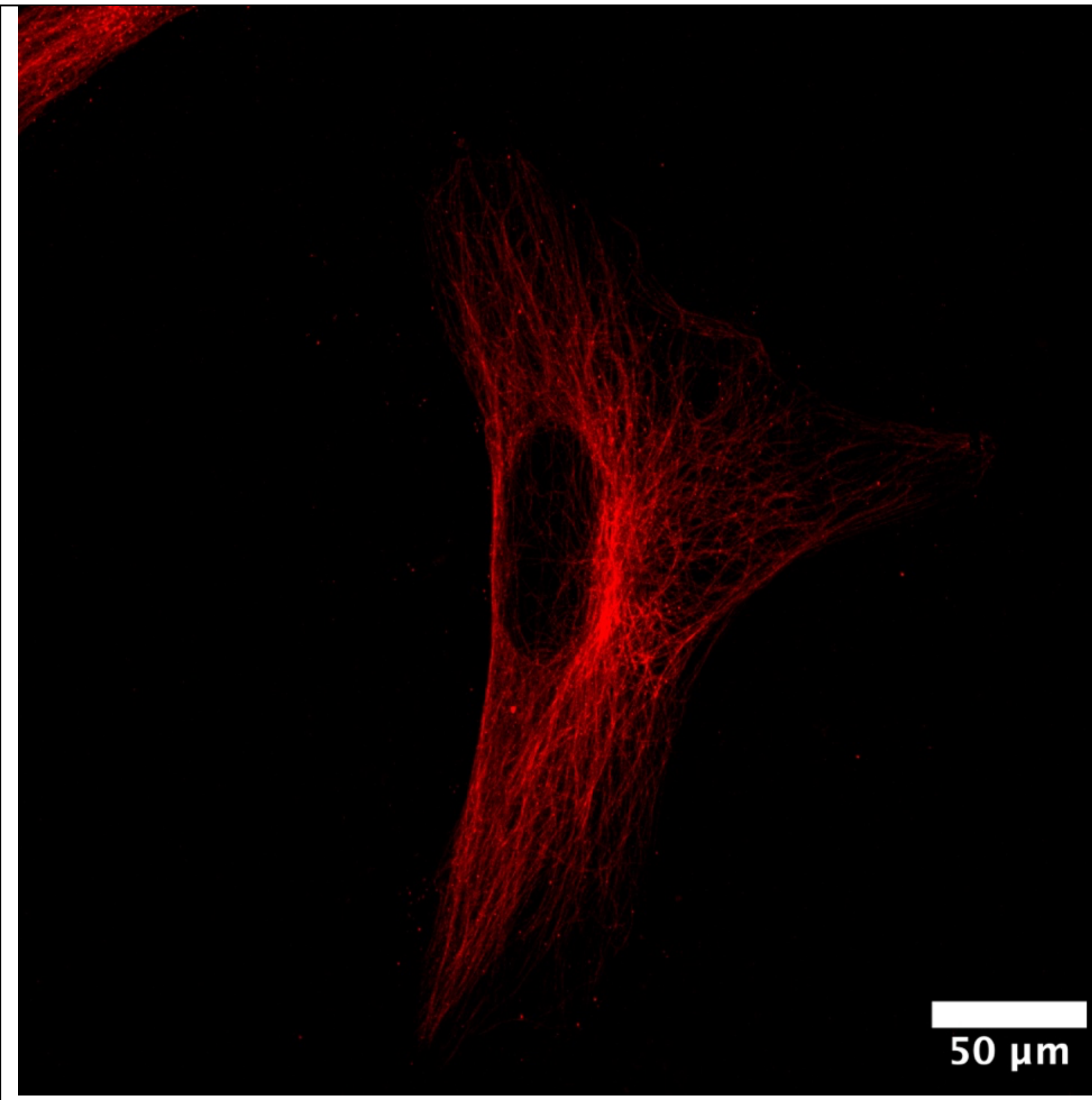
Arg-C	Promega	V1881	10 ug/mL<br>50 mM Tris, pH 7.8<br>2 mM EDTA, 10 mM DTT<br>37℃, 16h<br>Modified from protocol reference D.	Microtubules appeared discontinuous

				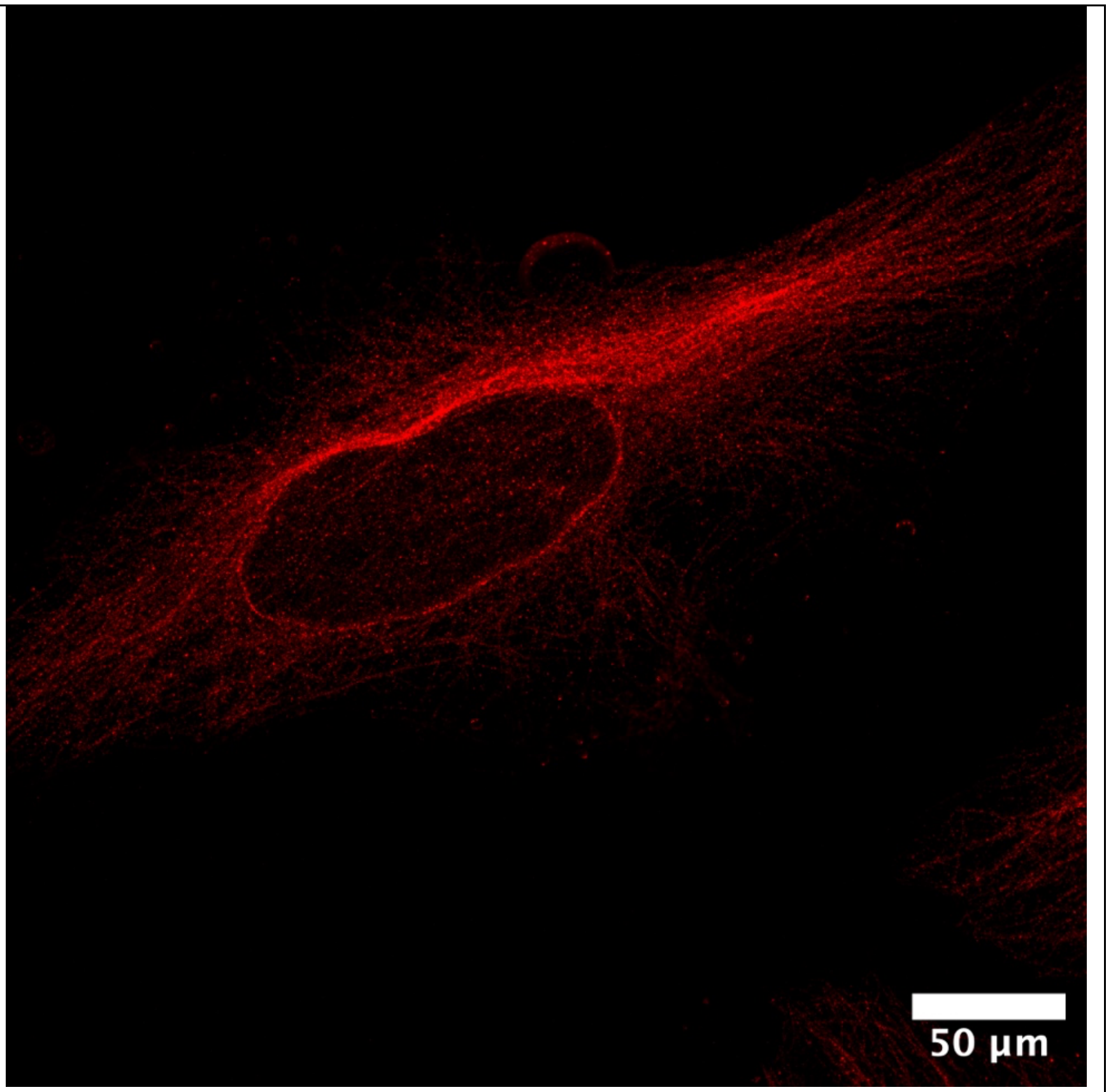
LysN	Promega	VA1180	20 ug/mL<br>50 mM Tris, pH 8<br>2 mM $ZnSO_4$<br>37℃, 16h	Cracks were observed within the gel & microtubules appeared discontinuous

				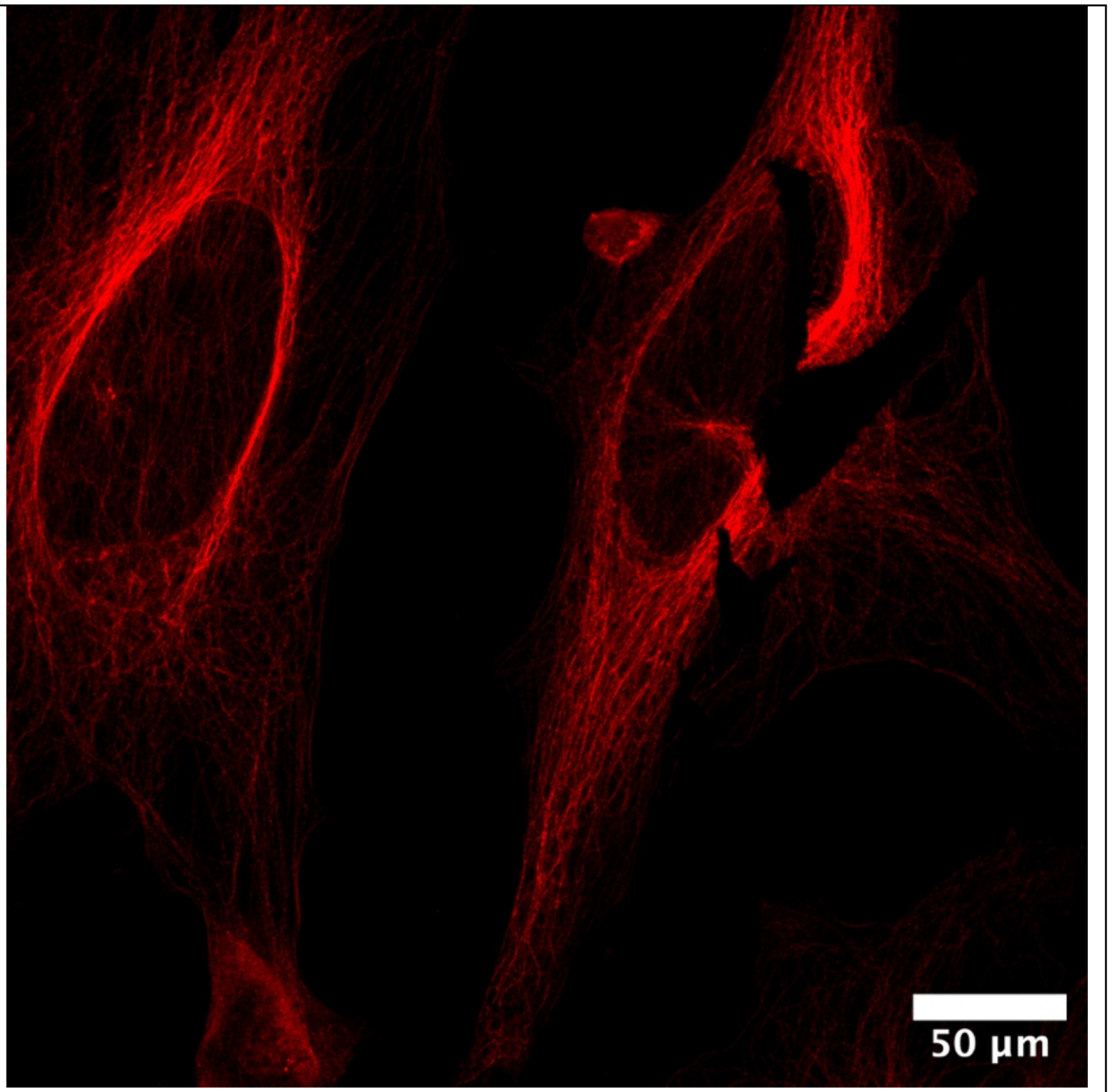
LysC/Trypsin	Promega	V5071	20 ug/mL<br>50 mM Tris, pH 8<br>37℃, 2h<br>Modified from protocol reference E.	Microtubules appeared discontinuous

				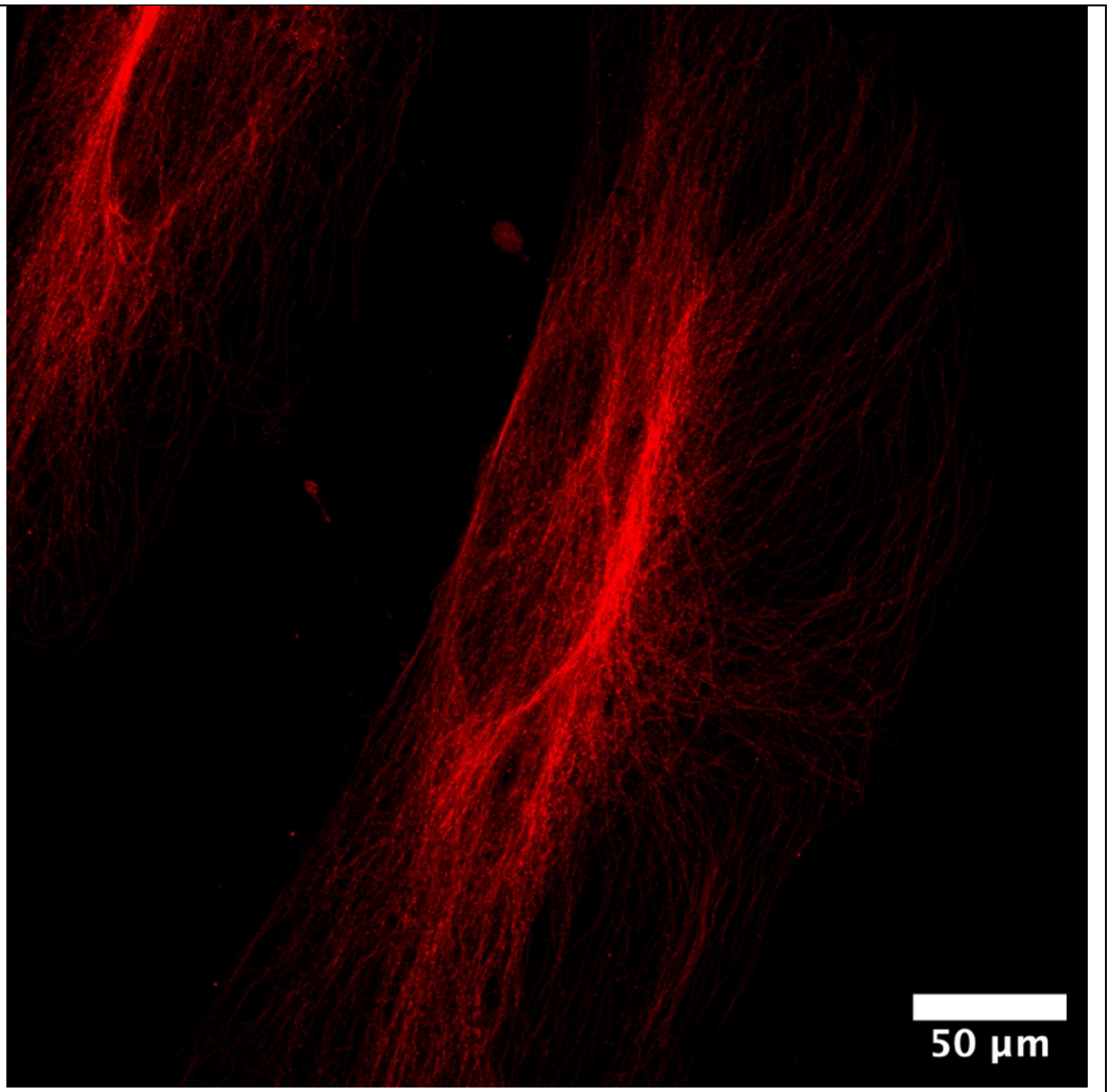
Chymotrypsin	Promega	V1061	25 ug/mL<br>100 mM Tris, pH 8<br>25℃, 16h<br>Modified from protocol reference F.	Cracks were observed within the gel & microtubules appeared discontinuous

				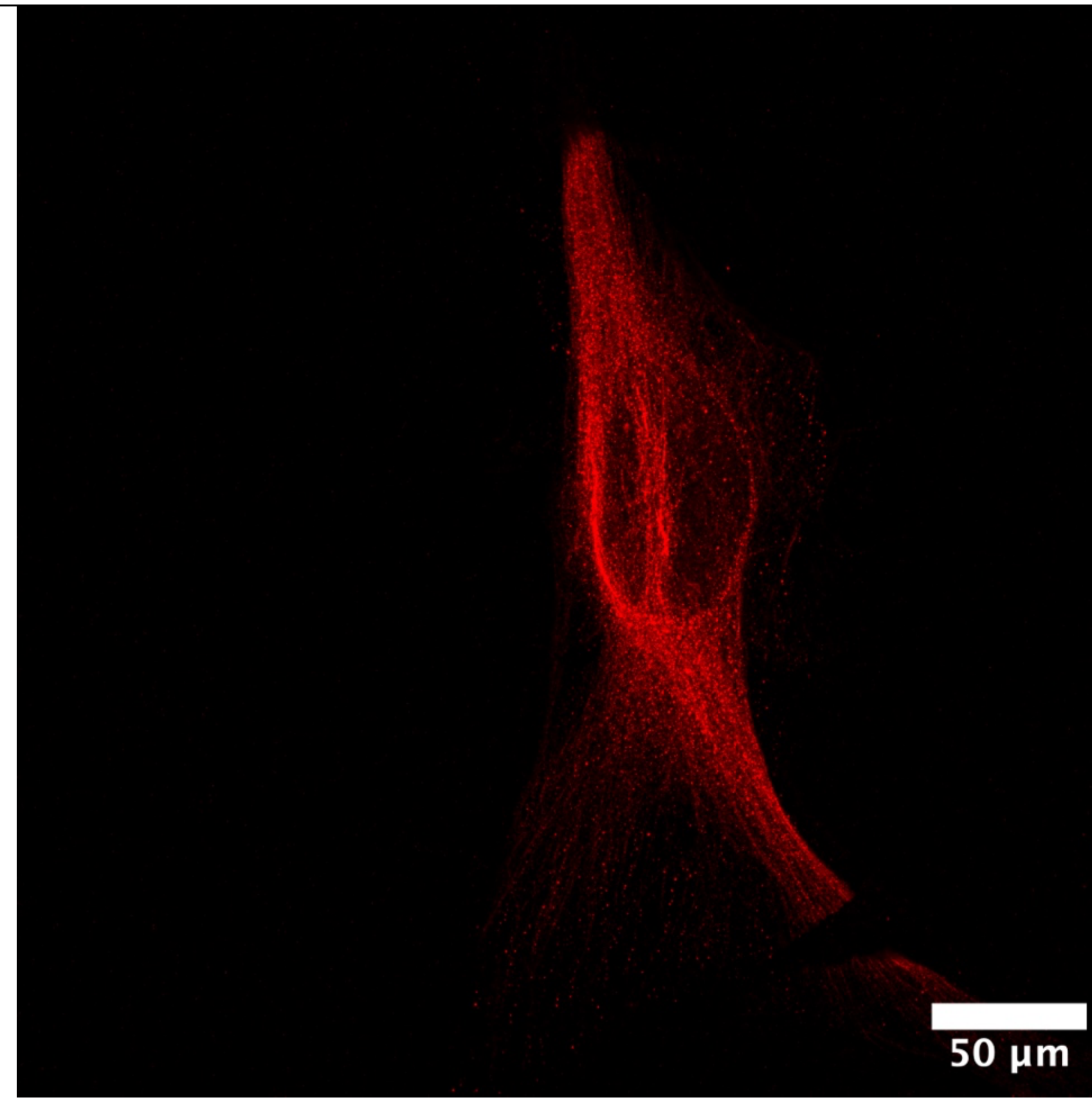 50 µm

Supplementary Table 2. Cleavable fixatives tested, and their outcomes.

Design Principles. We tried both custom-designed and commercially available cleavable crosslinking fixatives, guided by the following principles: first, the fixative molecule should be small enough to rapidly diffuse throughout the sample; second, the fixative should have multiple reactive groups that target functional groups on proteins (to achieve crosslinking); third, the fixative should be fast-acting to quickly lock biomolecules in place; and fourth, the crosslink should be cleavable. For the fixatives tested, this cleavability is afforded in one of three ways: (1) the cleavable bond is within the crosslinker itself and can later be broken by a cleavage reaction; (2) the cleavable bond is formed post-crosslinking and can later be broken by a cleavage reaction; or (3) the cleavable bond is formed at the conjugation between the crosslinker and the protein rather than in the crosslinker backbone itself.

In Vitro Screening. *First crosslinker choices:* At the beginning of the project, we first attempted to custom-synthesize a homofunctional traceless fixative (bi-APB; a molecule with boronic acid on both ends – see table below). Bi-APB forms protein crosslinks based on the formation of stable iminoboronates on both ends, which can be reversed by adding fructose, dopamine, or glutathione[3], leaving no trace on the proteins – hence, “traceless.” However, our mass spectrometry results showed no desired product formation. This may be because the dissociation constant K_d was too large, resulting in weak binding between proteins and this crosslinker[4]. We also analyzed this crosslinker by reacting it with protein and using an SDS-PAGE gel to assess the crosslinking degree of the reaction mixture by molecular weight (MW). After electrophoretic separation, the gels were imaged and quantified in Fiji/ImageJ using the Gel Analyzer tool to determine crosslinking degree. We categorized the imaged bands into two groups: first, the source band (i.e. the lowest MW band assuming no cleavage) and, second, the crosslinked bands (i.e. all bands of MW higher than the source band). Crosslinking degree (range: 0–100%) was computed as the summed integrated intensity of crosslinked bands divided by the summed intensity of all bands (crosslinked + source).

For these experiments, we desired to simulate the high protein density found in vivo (e.g., a typical protein concentration might be 20%-30% of cell volume in many cells[5]); thus, we tried to use a protein concentration of 100 mg/mL[6]. Specifically, to assess crosslinking degree for these early crosslinkers, we reacted 100 mg/mL BSA with 15 mM crosslinker in 50 mM phosphate buffer for 12 hr without shaking. Bi-APB still showed no crosslinking with these PAGE-gel based experiments.

Second set of crosslinkers: We then moved on to test cleavable crosslinkers, the rationale being that after protein crosslinking, we could cleave the cleavable bond in the crosslinker so that it would not prevent expansion in the later expansion step. We next tested 15 commercially-available crosslinkers. These crosslinkers were assessed for crosslinking degree by reacting with protein followed by gel electrophoresis using the same conditions as Bi-APB experiments: 100 mg/mL BSA with 15 mM crosslinker in 50 mM phosphate buffer for 12 hr without shaking.

Third set of crosslinkers: We also tested 7 custom-designed crosslinker candidates. For these crosslinkers, at the beginning of our design (see **Design Principles**), we had glutaraldehyde in mind but with a cleavable bond—and we wanted to compare to glutaraldehyde's crosslinking ability (both crosslinking degree and reaction speed).

We aimed to use the same crosslinking degree assessment conditions as the first two sets of crosslinker tests; however, when we reacted 1.1% w/v crosslinker Sulfonyl-bis-acetaldehyde with 100 mg/mL protein, the entire reaction mixture formed into a white, solid-like mass. The reaction mixture's consistency was such that it could not be pipetted and run on an SDS-PAGE gel. In light of this result, we adjusted the protein concentration for this third set of crosslinkers to 10 mg/mL. Specifically, we reacted 10 mg/mL α-Lactalbumin with 1.1% w/v crosslinker in 1× PBS for 1 hr without shaking.

A few notes on the differences between the first two sets, and the third set of crosslinkers, regarding assessment conditions: **Reaction Time:** We initially set the reaction time to 1 hr considering the timescale of conventional chemical covalent fixation using PFA or GA is about 10 minutes, but the specific choice of 1 hr was arbitrary. Additionally, if a reaction takes longer than 1 hr for a cultured cell, we reasoned that we would not want such a slow-reacting fixative, as it would not be quick enough to lock biomolecules in place and biomolecules might rearrange during this time. However, when we reacted first and second-set crosslinkers with protein, the crosslinking degree was very low (<10%), so we extended the reaction time to 12 hr for this subset of crosslinkers to better discriminate between their crosslinking efficacies, purely for chemical exploration. **Protein:** We initially utilized BSA for first and second-set tests as it is a standard laboratory protein product; however, we switched to alpha-Lactalbumin for third-set tests because the protein purity of that reagent was higher. **Reaction Buffer:** In initial tests with first and second-set crosslinkers, we used phosphate buffer without other salts because the dissolved salts might promote excessive protein precipitation when conducting the experiments. For third-set tests, the protein concentration was lower, and we desired to test in conditions as similar as possible to cell culture experiments, so PBS (phosphate buffer with salt) was used. **Crosslinker Concentration:** The first and second-set crosslinkers were primarily in a powder form so we used molar concentration (15 mM), third-set crosslinkers were primarily in a liquid form so we used mass fraction (1.1 % w/v).

For these reasons, the stated crosslinking degree values for $1^{st}/2^{nd}$ set crosslinkers should not be compared to third-set crosslinkers. Furthermore, we ultimately found that the *in vitro* PAGE-based crosslinking degree assessments did not correlate well to the results observed when crosslinkers were evaluated in cell culture (all of which gave poor results in cells).

In Situ Screening on HeLa Cells. Next, we applied cleavable fixative candidates to cultured HeLa cells. For fixation-only experiments (no ExM processing and no cleavage), cells were fixed under candidate-specific conditions, as indicated below each corresponding confocal image. Fixation-only immunostaining was performed as follows: cells were incubated in 200 µL of MAXblock Blocking Medium for 15 min at RT, followed by a single wash with MAXwash™ Washing Medium for 10 min at RT. For primary antibody staining, rabbit anti-tubulin (Abcam, catalog no. ab6046) was diluted 1:100 in MAXbind™ Staining Medium. Approximately 200 µL of the primary antibody solution was added to each well of a 24-well plate, followed by incubation for 2 h at room temperature. After primary antibody incubation, cells were washed three times with 1×PBS for 10 min each at room temperature. For secondary antibody staining, goat anti-rabbit Alexa Fluor 546 (Thermo Fisher Scientific, catalog no. A-11035) was diluted 1:100 in MAXbind™ Staining Medium. Approximately 200 µL of the secondary antibody solution was added to each well, followed by incubation for 2 h at room temperature. Cells were washed three times with 1×PBS for 10 min each at room temperature. Microtubule preservation was assessed qualitatively from confocal images by eye. Confocal overview images were acquired either using a 40× water-immersion objective, or a 10× air objective as indicated below the confocal images. In all cases, the lower panel shows a magnified crop from the corresponding upper image.

For crosslinkers used as fixatives followed by ExM processing, cells were gently rinsed twice with prewarmed (37°C) DPBS to remove any residual cell culture media. A fixative supplemented with 1% PFA or 3%PFA in PBS was then added (note: we initially tested 1% PFA as a starting point (the choice of 1% was arbitrary). However, for some fixatives, even supplemented with 1% PFA the preservation was still very poor (similar to the condition without PFA), so in some cases we increased the PFA concentration to 3%, **indicated below in the table**.), and cells were incubated for 8 hours at RT. Cells were subsequently washed three times with PBS, 5 minutes each at RT. After removing the PBS, cells were incubated in 0.1 mg/mL AcX in PBS for 2-3 hours at RT without shaking. After anchoring with AcX, the cells were washed three times with 1x PBS, 5 minutes each.

Gelation was performed using a proExM monomer solution consisting of 8.625% w/v sodium acrylate, 2.5% w/v acrylamide, 0.15% w/v N,N′-methylenebisacrylamide, and 2 M NaCl in 1× PBS. Polymerization was initiated by adding ice-cold stock solution of 10% w/v TEMED and 10% w/v APS to the ice-cold proExM monomer solution to a final concentration of 0.2% w/v TEMED and 0.2% w/v APS, with APS added last. The solution was added to the 24-well plate containing coverslips, and then incubated on ice for 3 min to permeate the specimen with monomers. The coverslip was then removed from the well and placed cell-side down onto ~27 µL of ice-cold monomer solution (containing TEMED and APS) deposited at the center of the gelation chamber at RT. Additional solution was added from the side to fill the chamber if necessary, and the coverslip was gently pressed to ensure contact with the spacers, yielding a uniform gel

thickness. Finally, the assembly was placed in a humidified box and incubated at 37°C for 1.5 h for gelation.

After the gelation process, samples were subjected to specific softening and cleavage protocols based on the fixative chemistry. Unless otherwise specified, all incubation steps involving shaking were performed on an orbital shaker at 50 rpm.

For fixatives with a sulfone bond, gels were incubated in a solution containing 8 M urea and 50 mM dithiothreitol (DTT) in water (adjusted to pH 9.3 with NaOH) for 20 minutes at RT with shaking. This incubation accomplished softening (via urea and DTT) and crosslinker cleavage (via high pH) in the same step. While standard protocols for sulfone-based crosslinkers utilize high alkaline conditions[7], we performed the reaction at a reduced pH of 9.3. This condition yielded sufficient cleavage to allow for expansion and might mitigate the risk of peptide bond cleavage associated with extreme alkalinity[8].

For fixatives with disulfide bond, gels were incubated in a solution consisting of 8M urea and 50 mM DTT in water (with no pH measurement or adjustment) for 20 minutes at RT with shaking. Since the crosslinkers' cleavable site is a disulfide moiety, the cleavage and softening actions are accomplished simultaneously by the urea+DTT solution.

For fixatives with diol bonds, gels were softened by incubation in a solution consisting of 8 M urea and 50 mM DTT in water (with no pH adjustment) for 20 minutes at RT with shaking, followed by three washes with 1x PBS for 30 minutes each at RT. The cleavage was then performed using a protocol adapted from[9]. Briefly, gels were incubated with 15 mM sodium periodate in 20 mM sodium phosphate buffer (pH 6.5) and 1M NaCl, for 30 min at RT, protected from light. The solution was then replaced with fresh 20 mM sodium phosphate buffer containing 15 mM sodium periodate, and incubation proceeded for an additional 4 hours protected from light.

For fixatives that bear two ester sites, gels were softened by incubation in a solution consisting of 8 M urea and 50 mM DTT in water (with no pH adjustment) for 20 minutes at RT with shaking, followed by three washes with 1x PBS for 30 minutes each. Cleavage was then performed by incubating gels with 0.5M hydroxylamine in 0.5M potassium phosphate buffer (pH 8.5) and 1M NaCl, incubating for 6 hr at 37 °C with shaking.

Following the specific cleavage step, all gels were washed three times with 1× PBS for 30 minutes each at RT.

For immunostaining, the gel was first incubated in 400 µL of MAXblock blocking medium for 30 minutes to 1 hour. This was followed by a single wash with MAXwash™ washing medium for 10 minutes to prepare the gel for antibody incubation. For primary antibody staining, rabbit anti-tubulin (abcam, catalog no. ab6046) was diluted 1:100 in MAXbind™

staining medium. Approximately 250 µL of the primary antibody solution was added to each well of a 24-well plate, and the gel was incubated for 5 hours at RT or 16h at 4°C. After primary antibody binding, the gel was washed three times with 1x PBS, 30 minutes each at RT. For secondary antibody staining, goat anti-rabbit AF546 (Thermo Fisher, catalog no. A-11035) was diluted 1:100 in MAXbind™ staining medium. Similar to the primary antibody step, 250 µL of the secondary antibody solution was added to each well, followed by incubation for 5 hours at RT or 16h at 4°C. The gel was washed three times with 1x PBS, 30 minutes each at RT. Finally, the gel was expanded in deionized water for two 30-minute sessions, followed by a 16h incubation at RT. The microtubules and gels were examined in confocal images by qualitative inspection, checking for cracking and microtubule continuity.

Vendor	Cat.#	Name	Chemical Structure	Cleavage	Results	Fixation results only, no ExM processing (The lower panel is a magnified crop from the corresponding upper image)
n.a. (custom designed)	n.a. (custom designed)	A1 (Sulfonyl-bis-acetaldehyde)	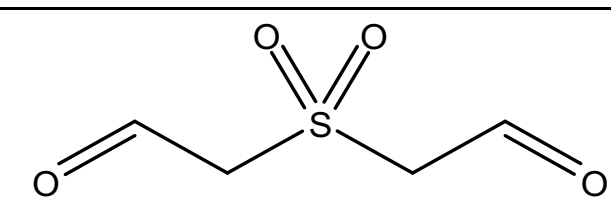	Bears a sulfone bond which can be cleaved by sodium hydroxide	Crosslinking degree: 86%<br><br>(When 1.1% w/v A1 reacted with 100 mg/mL purified α-Lactalbumin, the proteins became a white precipitate, therefore unable to run SDS-PAGE gel to assess the crosslinking degree. Therefore, we used 10 mg/mL protein for reaction.)<br><br>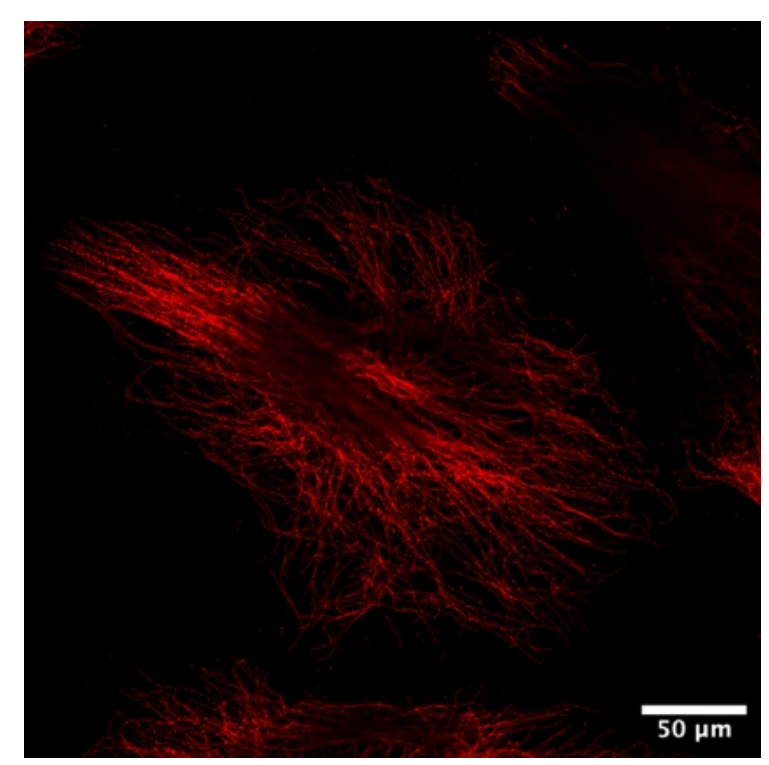	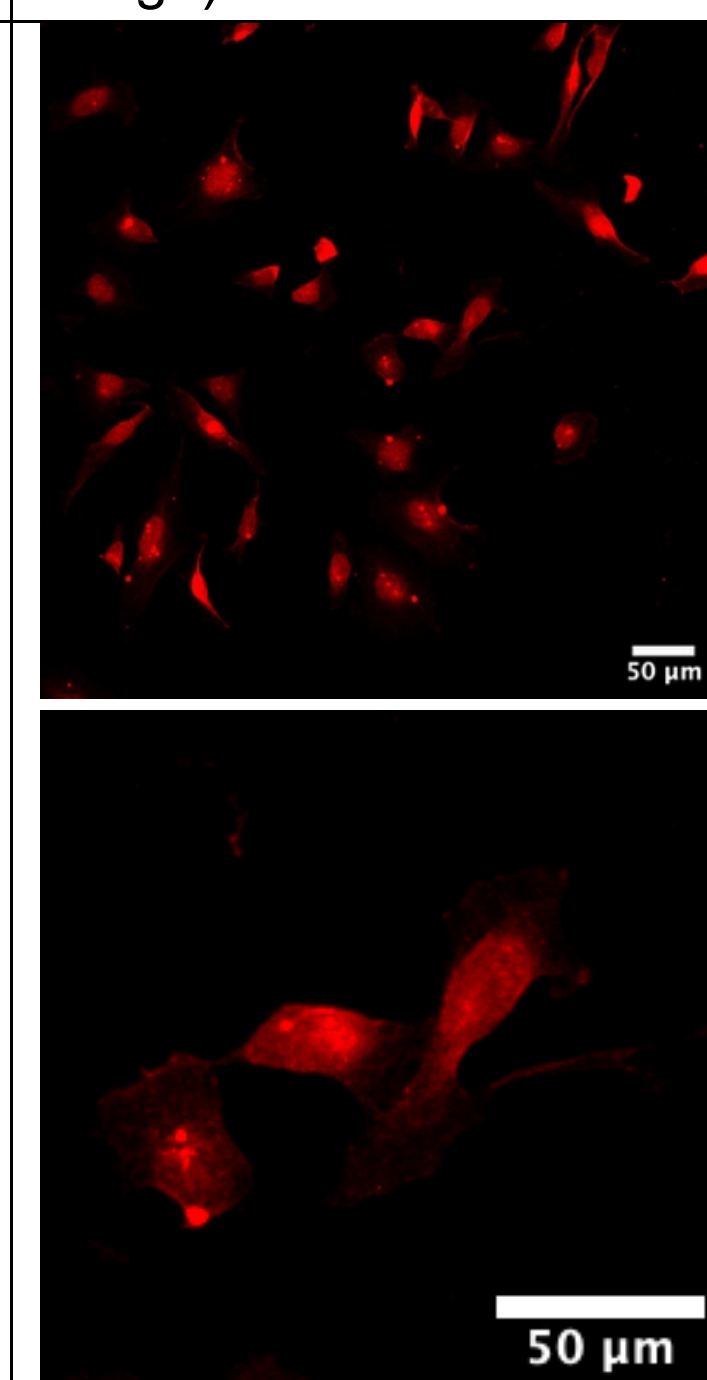<br>HeLa cells fixed with 4% v/v A1 in PBS, incubated for 6h at RT (fixation only, no ExM), and stained against beta-tubulin. The

					4% v/v A1 supplemented with 1% w/v PFA in PBS, 8h at RT, then post-expansion stained against beta-tubulin. The confocal image was acquired using a 40× water-immersion objective. Brightness and contrast settings: first set by the ImageJ auto-scaling function and then manually adjusted to improve contrast for the stained structures of interest (same for all the other images in Supp Table 2, except images without raw data)<br><br>Microtubules appear discontinuous after expansion	confocal image was acquired using a 10× air objective.<br><br>The cytoskeletal structure was almost completely gone.

n.a. (custom designed)	n.a. (custom designed)	A2 (2,2'-disulfanediyldiacetaldehyde)	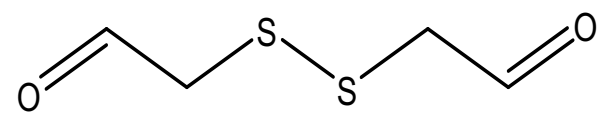	Bears a disulfide bond that can be cleaved by reduction (e.g. dithiothreitol)	Crosslinking degree: 7%<br>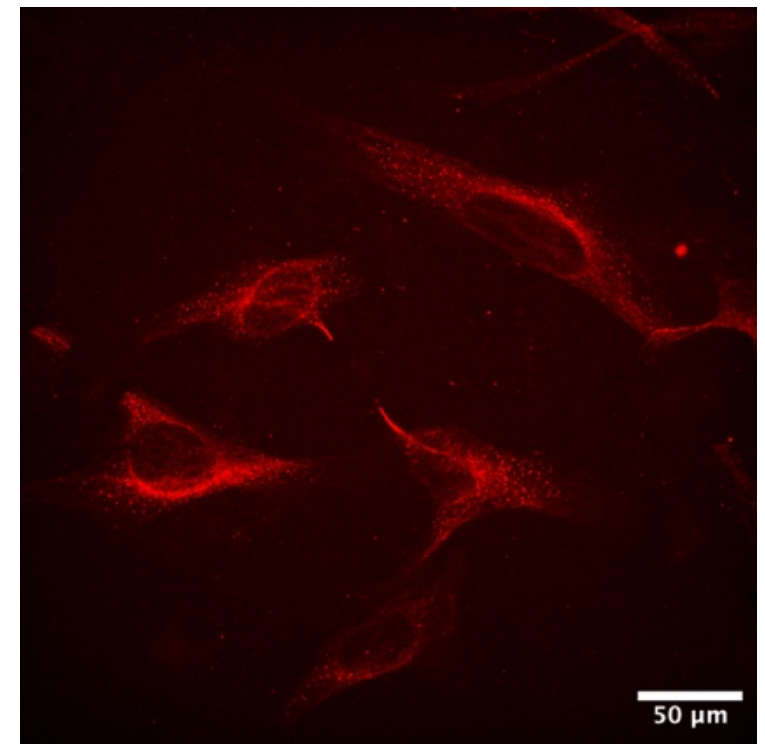<br>4% v/v A2 supplemented with 1%w/v PFA in PBS, 8h at RT, then stained against beta-tubulin. The confocal image was acquired using a 40× water-immersion objective.<br><br>Microtubules appear discontinuous	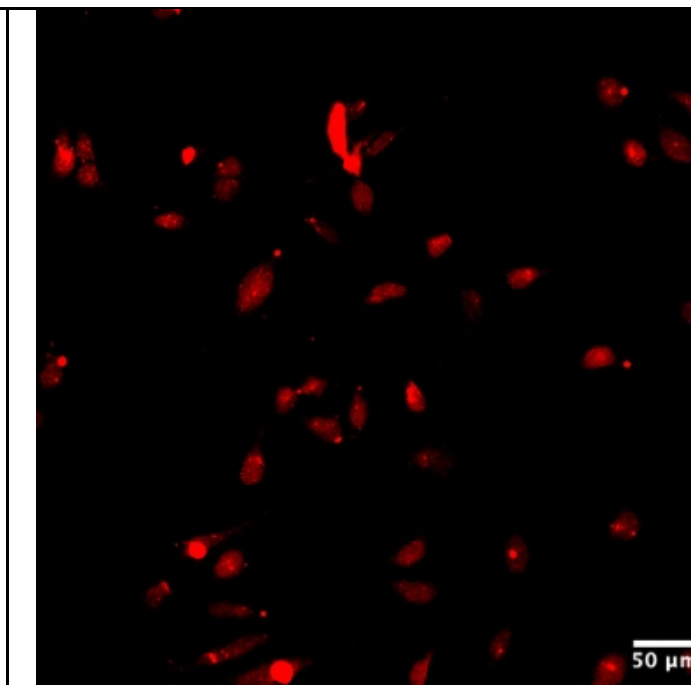<br>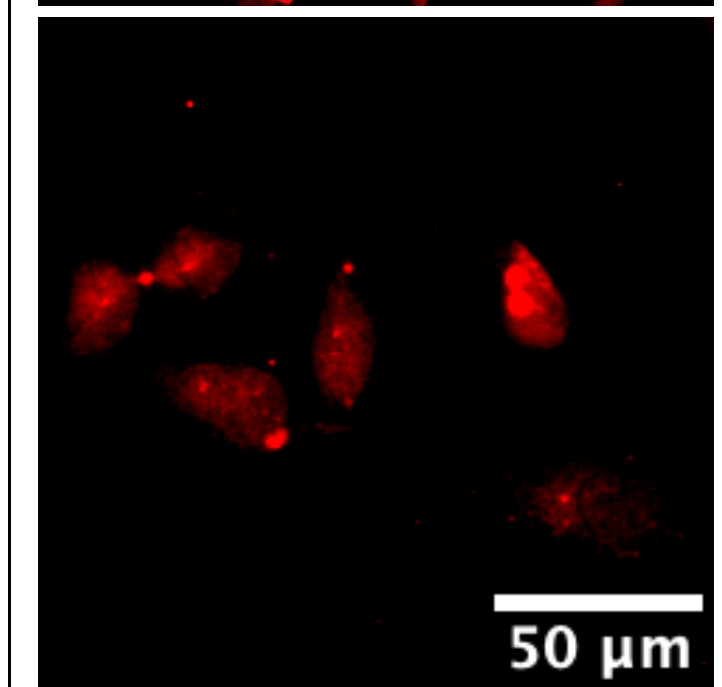<br>HeLa cells fixed with 4% v/v A2 in PBS, 6h at RT (fixation only, no ExM), and stained against beta-tubulin. The confocal image was acquired using a 10× air objective.<br><br>The cytoskeletal structure was almost completely gone.

n.a. (custom designed)	n.a. (custom designed)	CL1 (tartaric aldehyde)	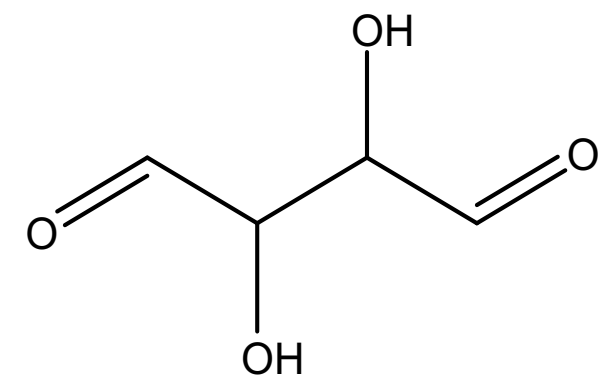	Bears a diol bond that can be cleaved by oxidation (e.g. sodium periodate)	No crosslinking (PAGE gel result)	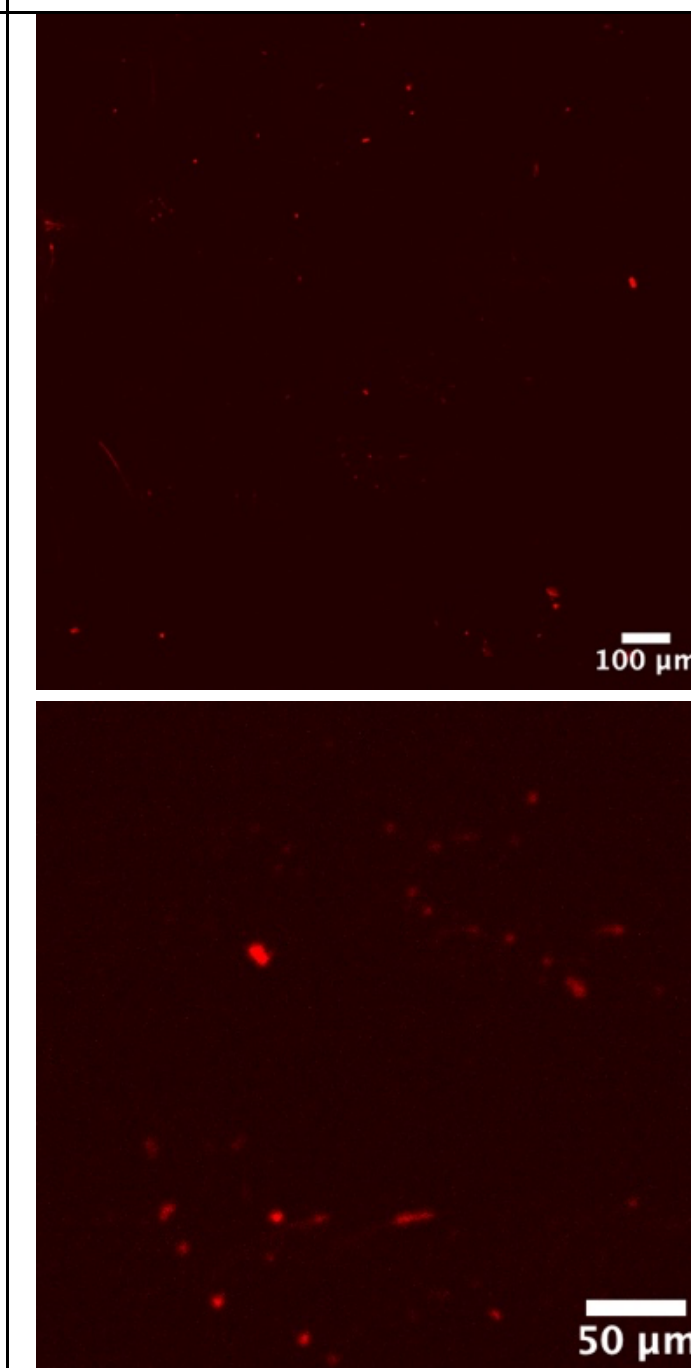 HeLa cells fixed with 4% v/v CL1 in PBS, 6h at RT (fixation only, no ExM), and stained against beta-tubulin. The confocal image was acquired using a 10× air objective.<br><br>No tubulin signal detected

n.a. (custom designed)	n.a. (custom designed)	CL2 (bis(chlorocarbonyl)(carbamoyl)disulfane)	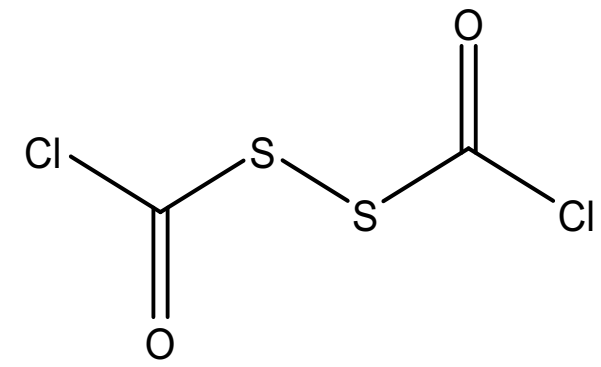	Bears a disulfide bond that can be cleaved by reduction	No crosslinking (PAGE gel result)	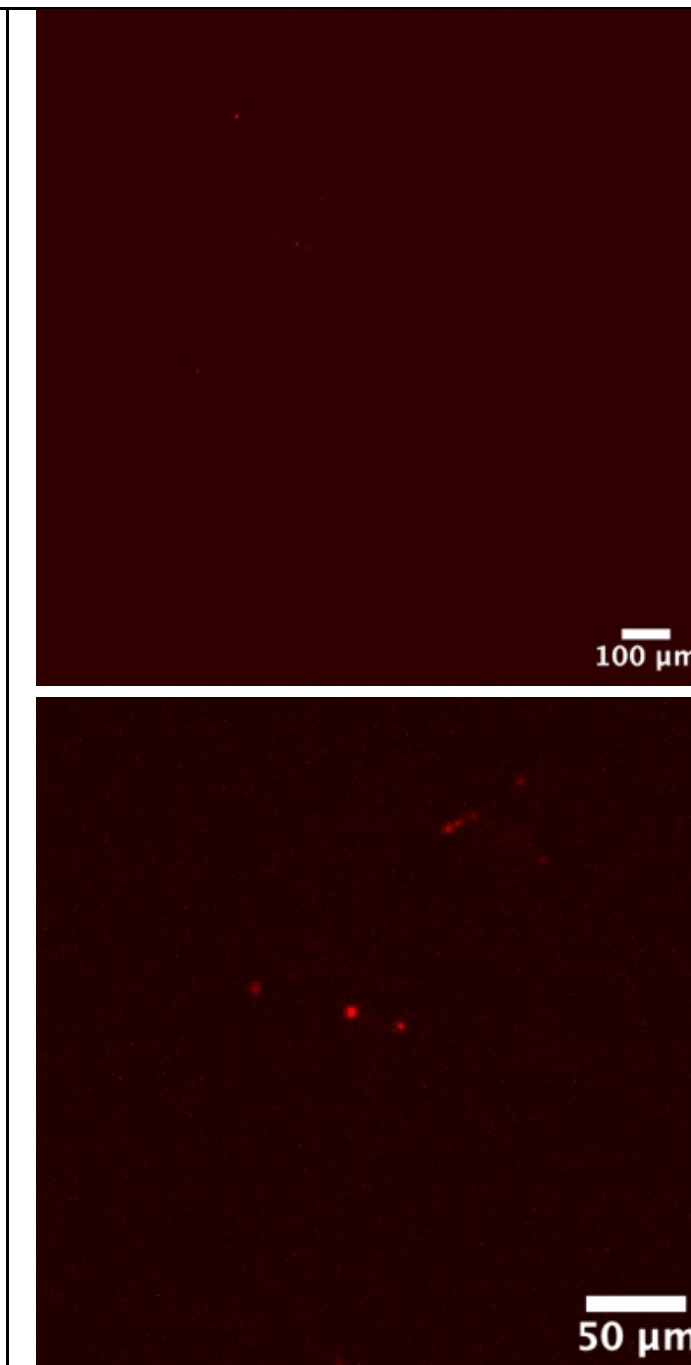 HeLa cells fixed with 4% v/v CL2 in PBS, 6h at RT (fixation only, no ExM), and stained against beta-tubulin. The confocal image was acquired using a 10× air objective.<br><br>No tubulin signal detected

n.a. (custom designed)	n.a. (custom designed)	B1 (2,2'-(sulfonylbis(methylene))bis(oxirane))	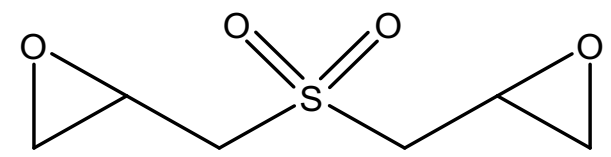	Bears a sulfone bond which can be cleaved by sodium hydroxide	Crosslinking degree 4%<br><br>Efficient epoxy-based crosslinking generally requires alkaline conditions, as epoxide groups react efficiently with protein sulfhydryl, amine, and hydroxyl nucleophiles only at elevated pH [10]. At near-neutral pH, their reactivity toward protein nucleophiles is low and intermolecular reactions with proteins proceed very slowly [11,12], so only long incubations substantially increase crosslinking in vitro, which limits their straightforward use in live-cell applications.	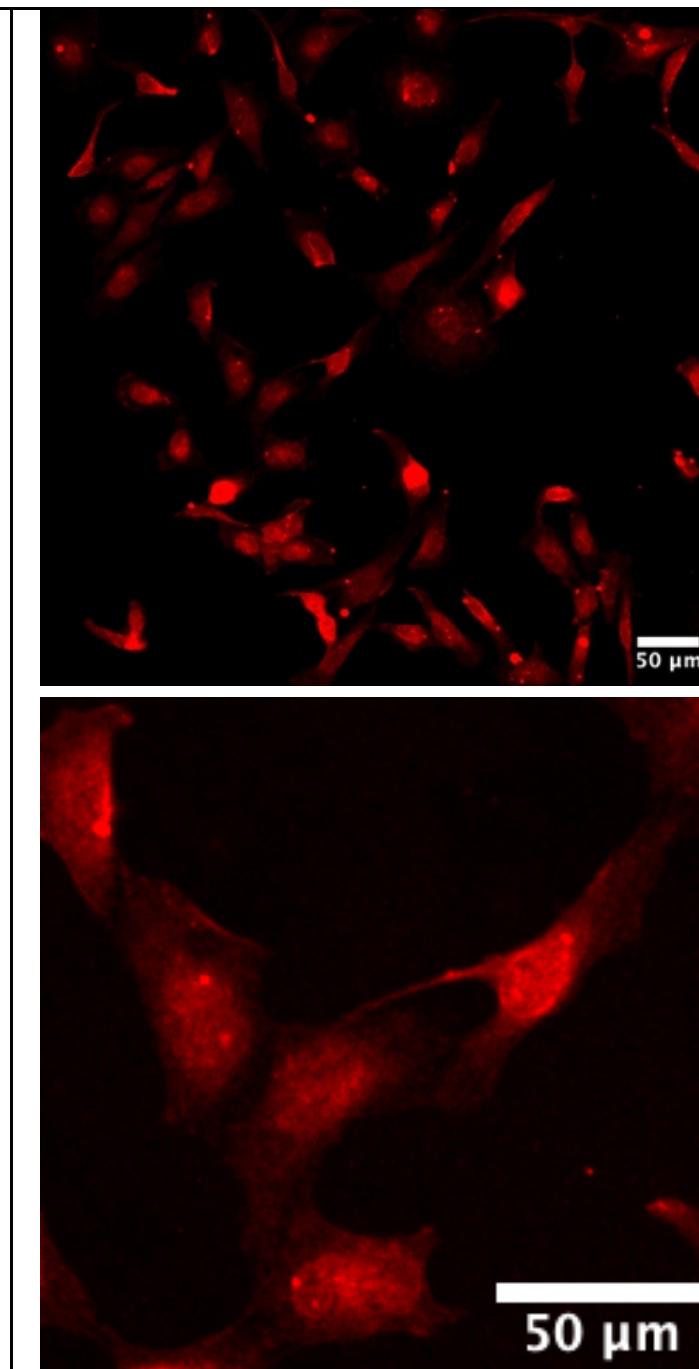 HeLa cells fixed with 4% v/v B1 in PBS, 6h at RT (fixation only, no ExM), and stained against beta-tubulin. The confocal image was acquired using a 10× air objective.<br><br>Tubulins were dispersed.

n.a. (custom designed)	n.a. (custom designed)	B2 (1,2-bis(oxiran-2-ylmethyl)disulfane)	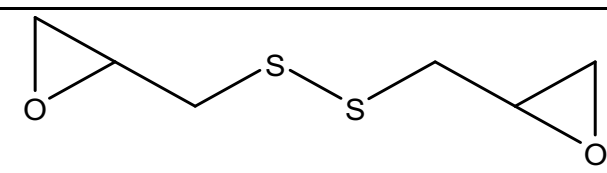	Bears a disulfide bond that can be cleaved by reduction	Crosslinking degree = 11%<br>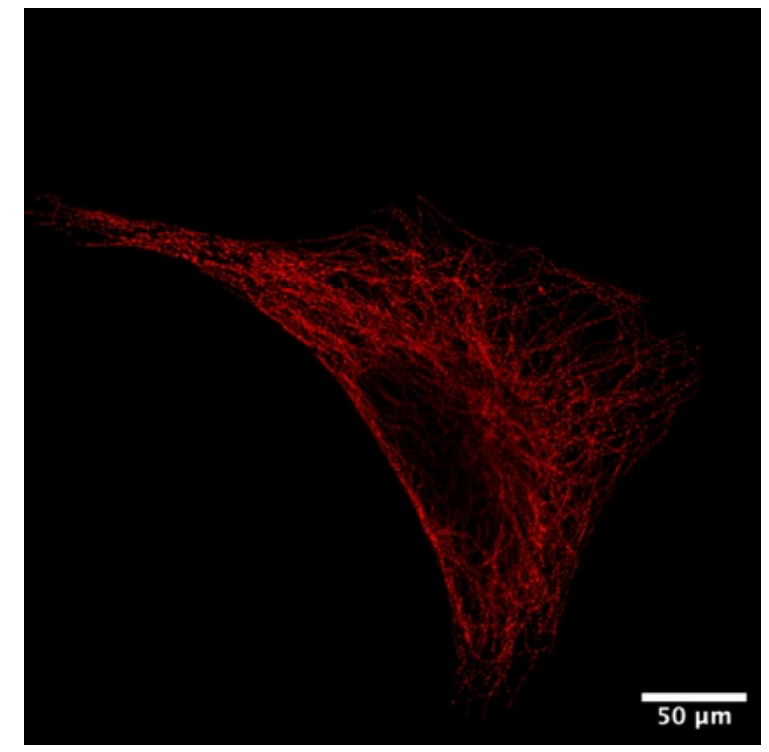<br>4% v/v B2 supplemented with 3%PFA in PBS, 8h at RT, then stained against beta-tubulin. The confocal image was acquired using a 40× water-immersion objective.<br><br>Microtubules appear discontinuous<br><br>Note: We aimed to minimize crosslinking between proteins; therefore, we initially tested 1% PFA as a starting point (the choice of 1% was arbitrary). However, preservation was	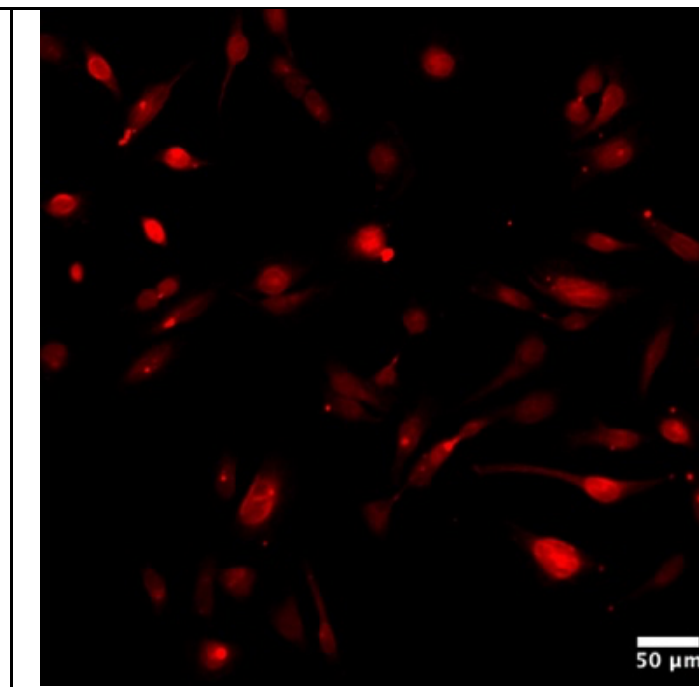<br>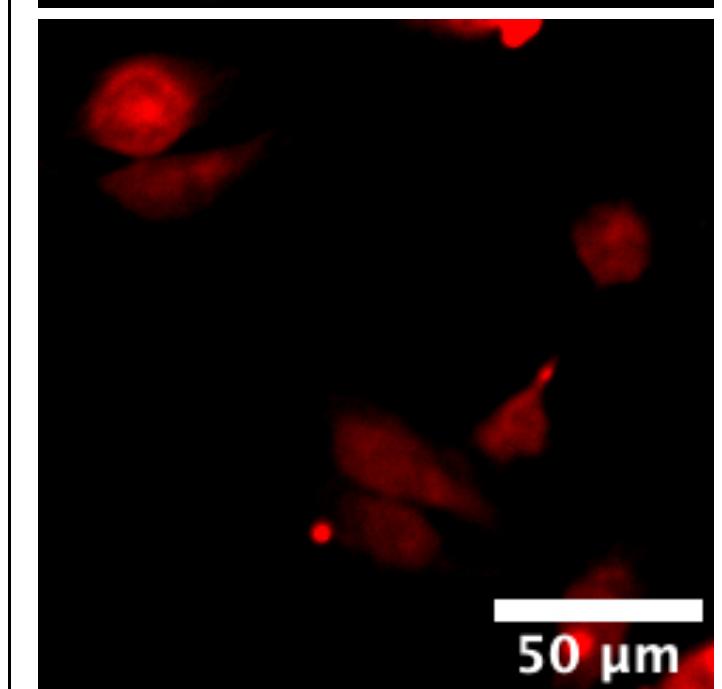<br>HeLa cells fixed with 4% v/v B2 in PBS, 6h at RT (fixation only, no ExM), and stained against beta-tubulin. The confocal image was acquired using a 10× air objective.<br><br>The cytoskeletal structure was almost completely gone.

					very poor (similar to the condition without PFA), so we increased the PFA concentration to 3%. The same applies to all subsequent conditions where 3% PFA was used instead of 1% PFA.	
n.a. (custom designed)	n.a. (custom designed)	B3 (1,2-di(oxiran-2-yl)ethane-1,2-diol)	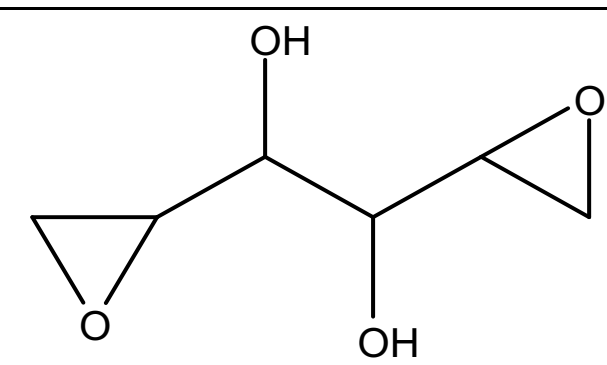	Bears a diol bond that can be cleaved by oxidation	Crosslinking degree: 4%<br><br>Note: For all crosslinkers with diol bonds (regardless of whether they are within the crosslinker or formed upon crosslinking), we qualitatively observed that after cleavage, the tubulin signal appears aggregated into clusters. This is likely because after cleavage, aldehyde groups are generated, which subsequently acts as new crosslinkers. This interpretation is consistent with the known periodate-mediated oxidation/cleavage of vicinal diols to aldehydes, which	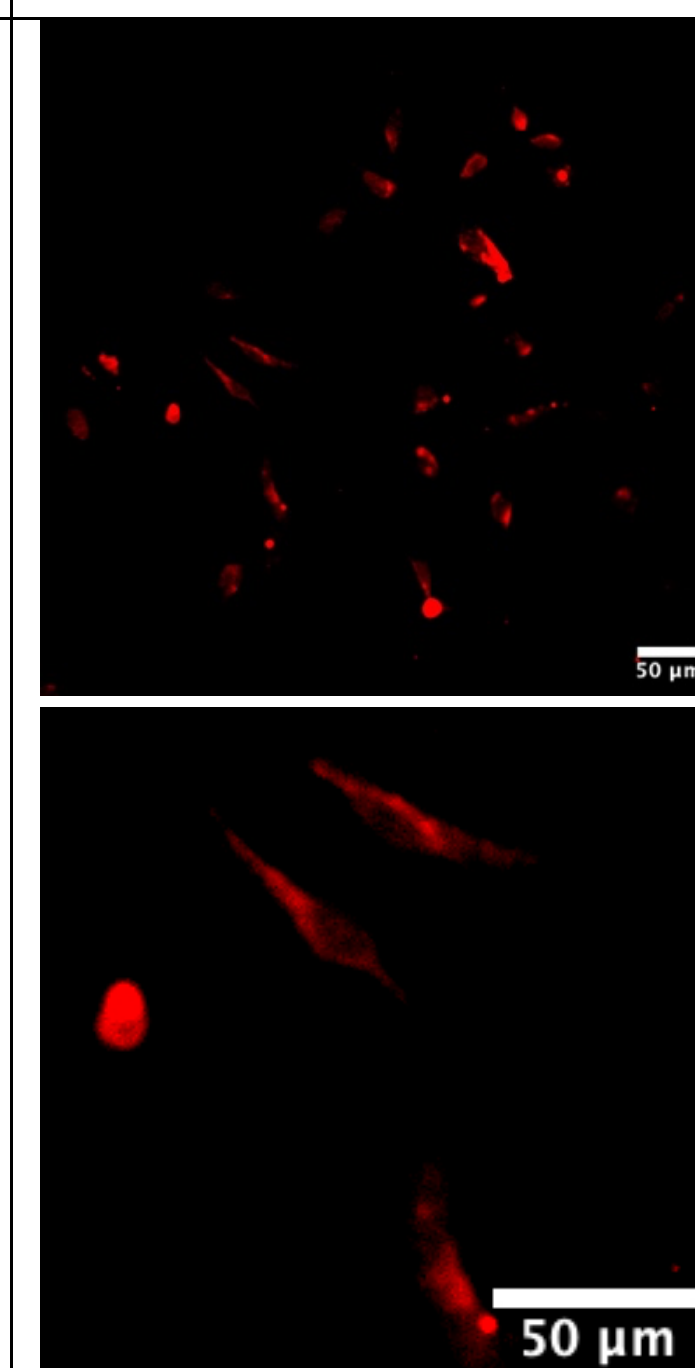 HeLa cells fixed with 4% v/v B3 in PBS, 6h at RT (fixation only, no ExM),

					can subsequently form additional covalent bonds with nucleophiles such as amines and are exploited in periodate–lysine–paraformaldehyde (PLP) fixation protocols [13].	and stained against beta-tubulin. The confocal image was acquired using a 10× air objective.<br><br>Tubulins were dispersed.
n.a. (custom designed)	n.a. (custom designed)	Traceless crosslinker Bi-APB	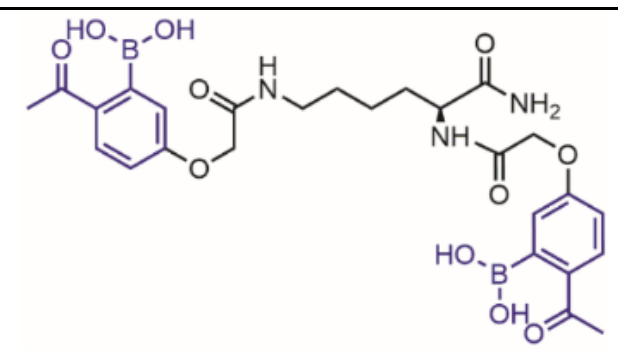	The reaction between amines and boronic acids is reversible [3]	No crosslinking (PAGE gel result)	Not tried on cells
Sigma Aldrich	202533	DEB (1,3-Butadiene diepoxide)	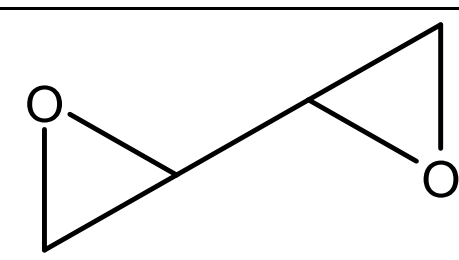	Bears no cleavable bond but a cleavable diol bond will form upon crosslinking	Crosslinking degree: 6%<br>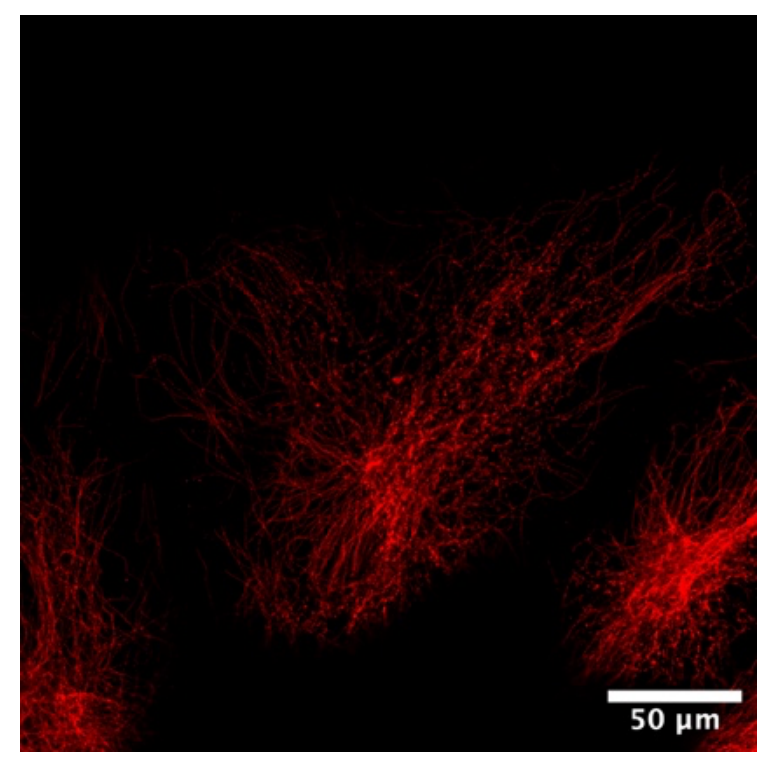<br>4% v/v DEB supplemented with 1% w/v PFA in PBS, 8h at RT, then stained against beta-tubulin post-expansion staining. The confocal	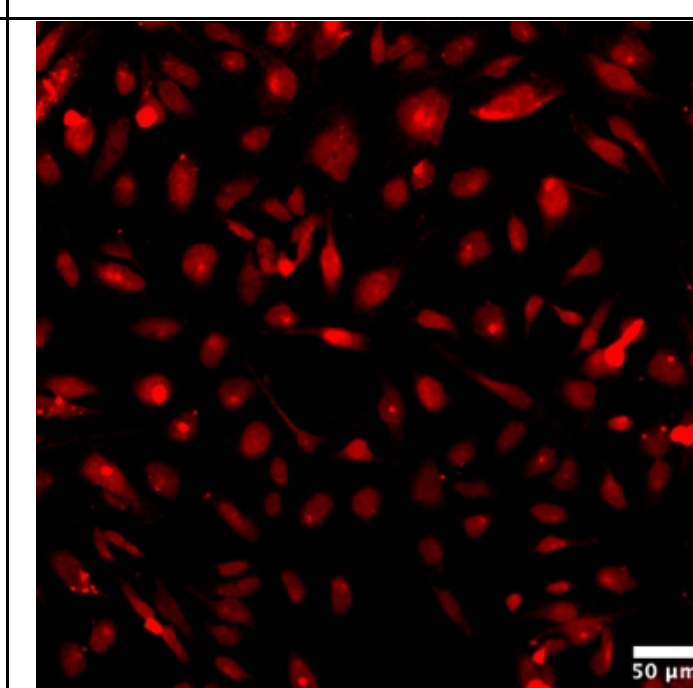

					image was acquired using a 40× water-immersion objective.<br><br>Microtubules appear discontinuous after expansion, and tubulin seems aggregated in some areas, possibly because after diol cleavage, new aldehyde groups are generated, leading to protein crosslinking and aggregation.	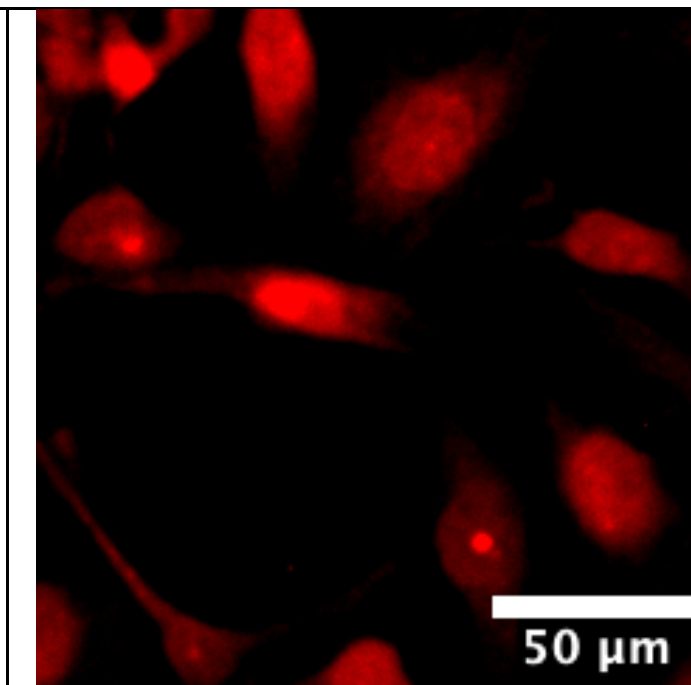<br>HeLa cells fixed with 4% v/v DEB in PBS, 6h at RT (fixation only, no ExM), and stained against beta-tubulin<br><br>Tubulins were dispersed.
ThermoFisher	22585	DSP (dithiobis(succinimidyl propionate))	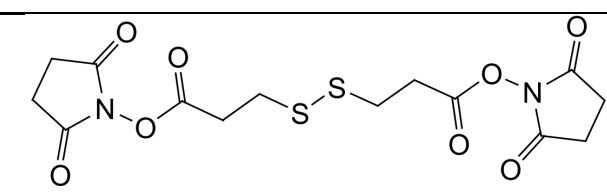	Bears a disulfide bond that can be cleaved by reduction	Crosslinking degree: 62 %<br><br>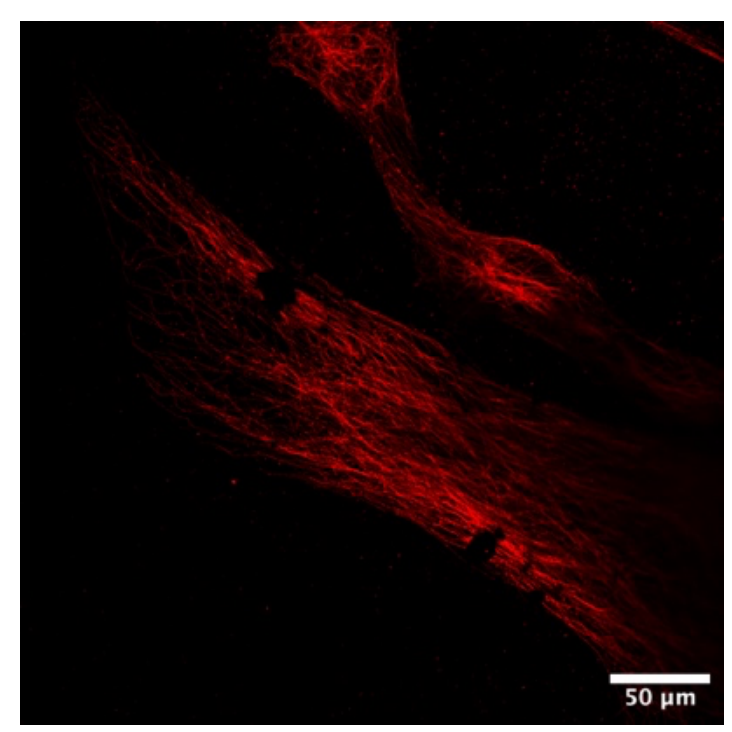<br>12.5 mM DSP supplemented with 1% w/v	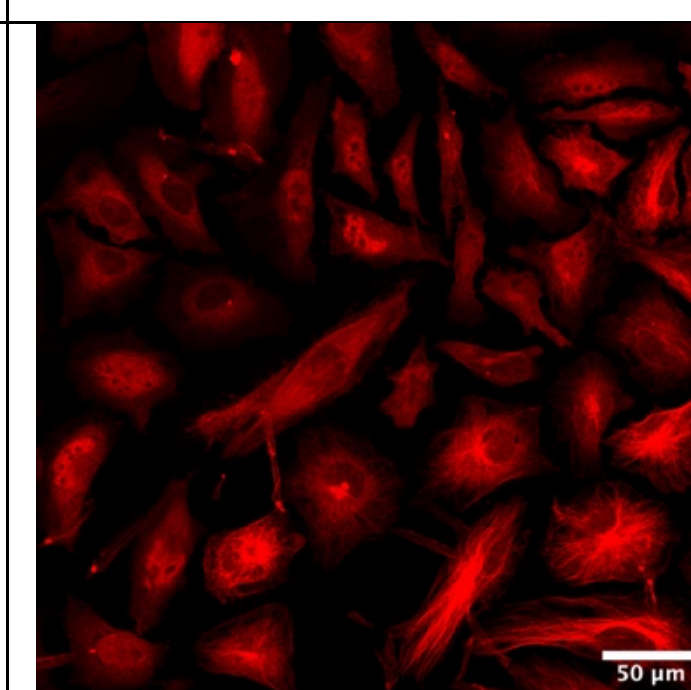

					PFA in PBS, 8h at RT, then stained against beta-tubulin. The confocal image was acquired using a 40× water-immersion objective.<br><br>Microtubules appear discontinuous and have cracks within the structure.	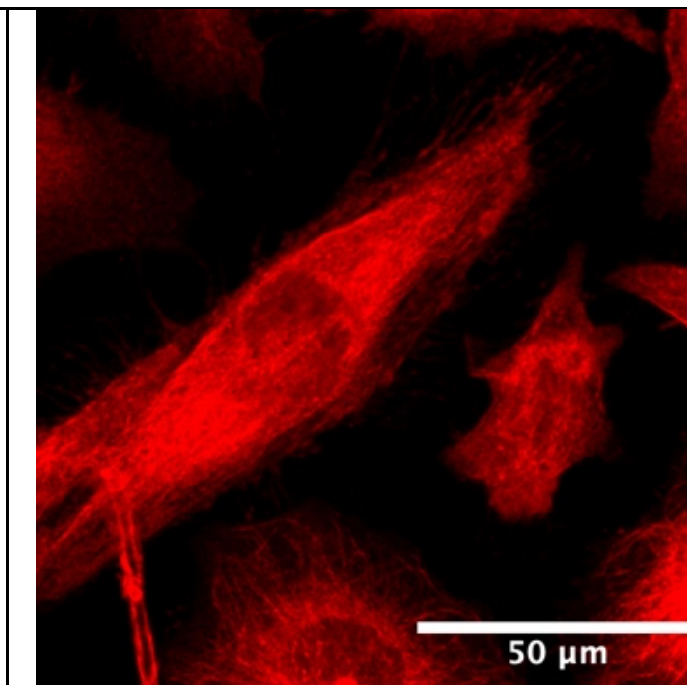<br>HeLa cells fixed with 20 mM DSP in phosphate buffer (pH 8), 6 h at RT (fixation only, no ExM), and stained against beta-tubulin.<br><br>Tubulins were dispersed.
ThermoFisher	20589	DST (disuccinimidyl tartrate)	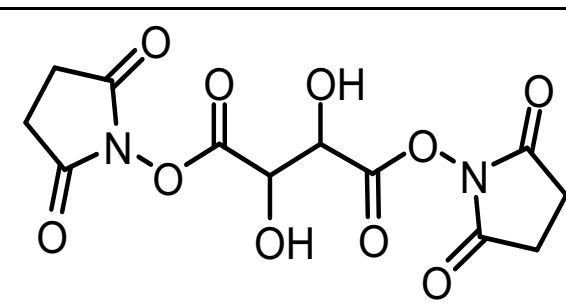	Bears a diol bond that can be cleaved by oxidation	Crosslinking degree: 50%	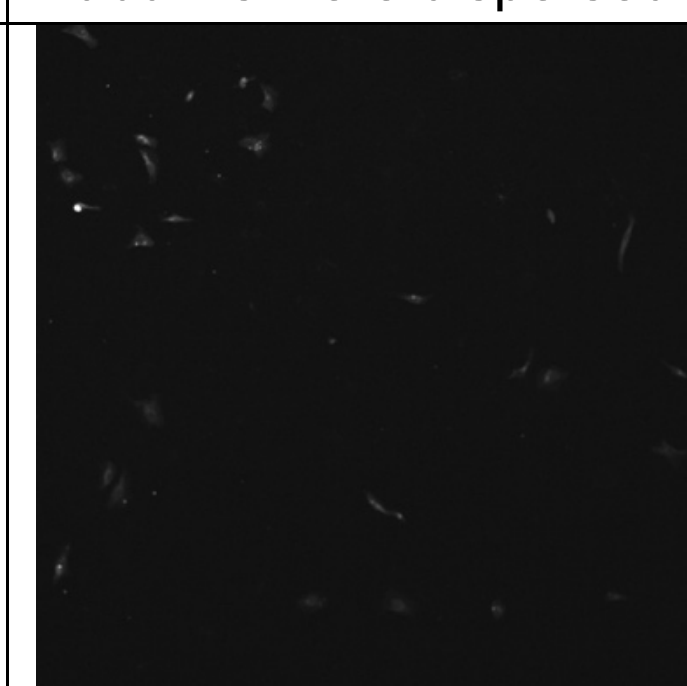

						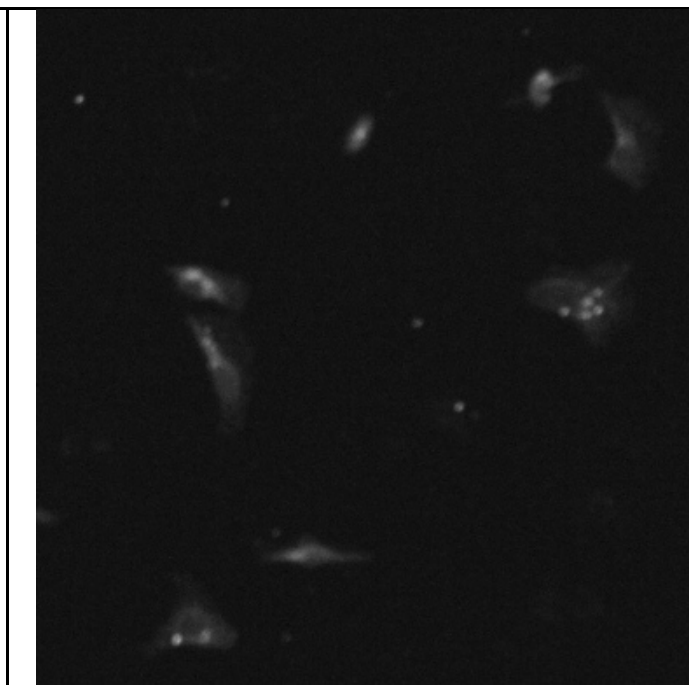<br>HeLa cells were fixed with 20 mM DST in PBS for 6 hours at RT, then stained against beta-tubulin. The confocal image was acquired using a 10× air objective.<br><br>Tubulins were dispersed.<br><br>Raw confocal files were unavailable; therefore, scale bars are not shown.
ThermoFisher	21600	BSOCOES (bis[2-(succinimidyloxycarbonylox	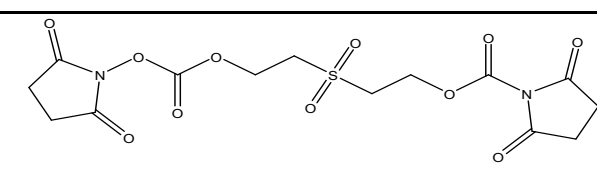	Bears a sulfone bond which can be cleaved by sodium hydroxide	Crosslinking degree: 75 %	

		y)ethyl] sulfone)			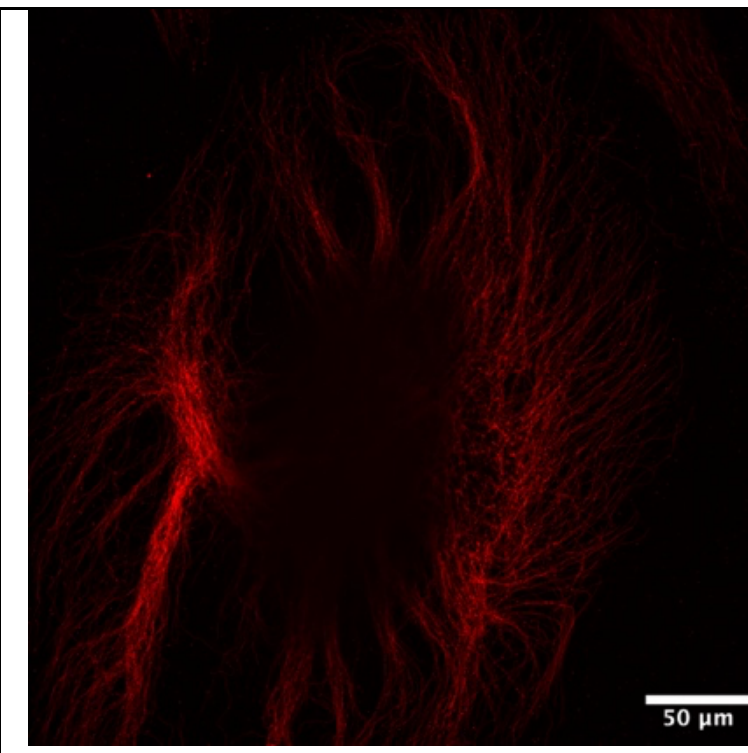<br>12.5 mM of BSOCOES supplemented with 1% w/v PFA in PBS, 8h at RT, then post expansion stained against beta-tubulin. The confocal image was acquired using a 40× water-immersion objective.<br><br>Microtubules appear discontinuous	
ThermoFisher	21565	EGS (ethylene glycol bis(succinimidyl	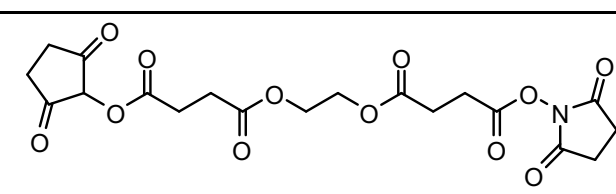	Bears two ester sites can be cleaved with hydroxylamine	Crosslinking degree was not recorded	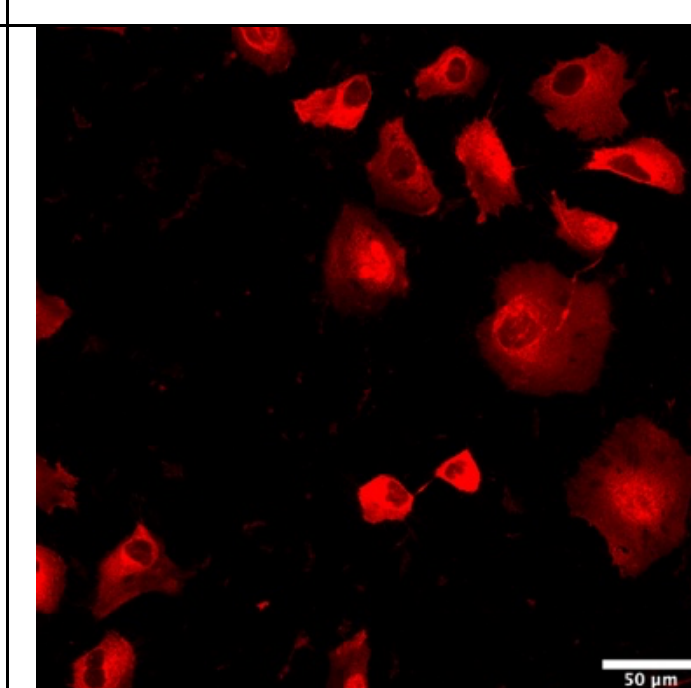

		succinate))				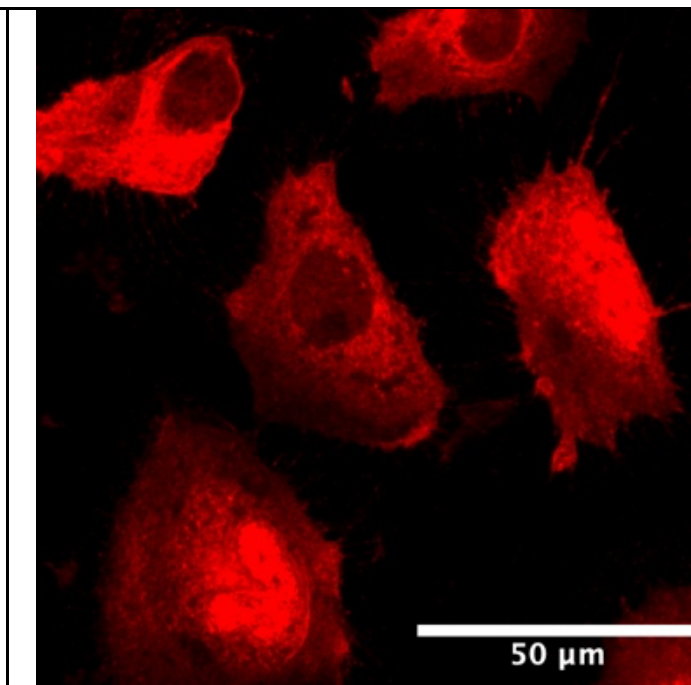HeLa cells were fixed with 6.25 mM EGS + 1% PFA, in PBS, incubated for 6h at RT (fixation only, no ExM), and stained against beta tubulin. The confocal image was acquired using a 40× water-immersion objective.<br><br>Tubulins were dispersed.

ThermoFisher	21566	Sulfo-EGS (ethylene glycol bis(sulfosuccinimidyl succinate))	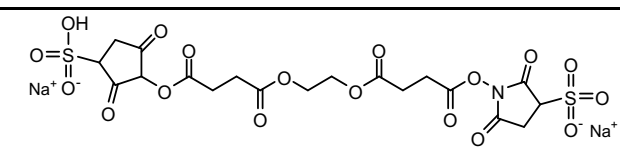	Bears two ester sites can be cleaved with hydroxylamine	Crosslinking degree: 19%	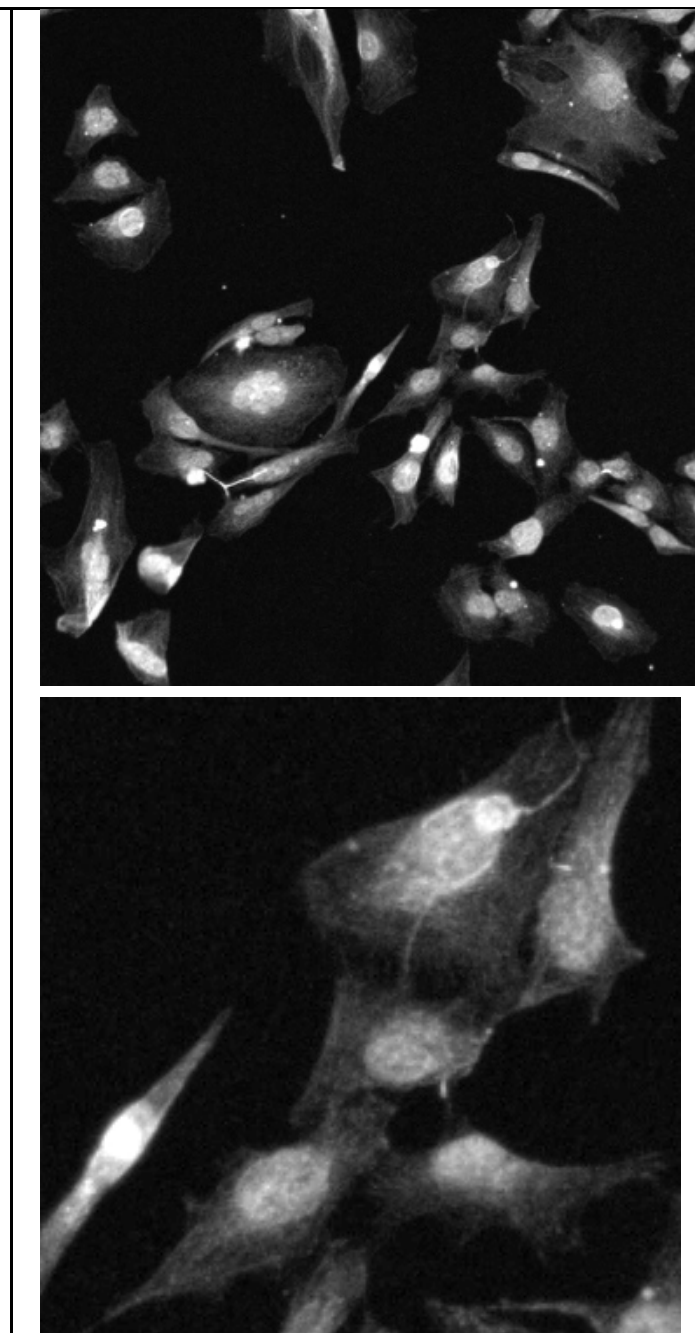 HeLa cells were fixed with 6.25 mM Sulfo-EGS + 1% PFA, in PBS, incubated for 6h at RT (fixation only, no ExM), and stained against beta tubulin. The confocal image was acquired using a 10× air objective.<br><br>Tubulins were dispersed.

						Raw confocal files were unavailable; therefore, scale bars are not shown.
ThermoFisher	21578	Sulfo-DSP (3,3'-dithiobis(sulfosuccinimidyl propionate))	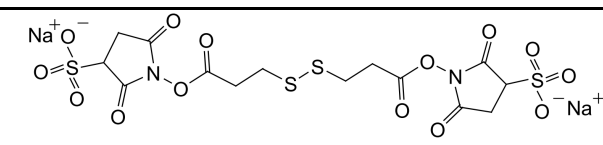	Bear a disulfide bond that can be cleaved by reduction	Crosslinking degree: 60%	We did not test this in cells because sulfo-DSP also bears a disulfide bond, like DSP, and we found that the cleavage efficiency of DSP was not high enough to give us a crack-free gel.
ThermoFisher	26169	SDAD (NHS-SS-Diazirine)	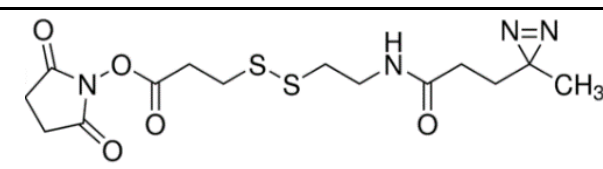	Bears a disulfide bond that can be cleaved by reduction	Crosslinking degree: 73%	Not evaluated in cells. At the time of the initial screen, we had not established whether the photoactivation required for SDAD-based fixation would preserve cellular biomolecules, including nucleic acids. We therefore did not advance these candidates to cellular testing. During subsequent development of nfExM, we adopted a brief, UV exposure for the separate purpose of initiating rapid hydrogel

						polymerization at −20°C, where APS/TEMED was ineffective. Photoinitiated gelation has previously been demonstrated in PhotoExM[14]. The effects of the nfExM irradiation condition on DNA and RNA integrity were not assessed.
ThermoFisher	26175	Sulfo-SDAD (Sulfo-NHS-SS-Diazirine)	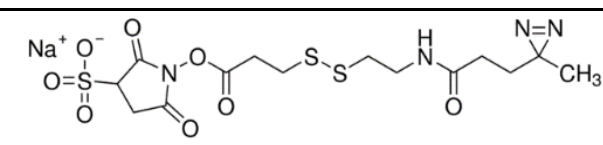	Bears a disulfide bond that can be cleaved by reduction	Crosslinking degree: 66%	Not evaluated in cells. See previous row for reasoning.
ProteoChem	c1116	SPDP (succinimidyl 3-(2-pyridyldithio) propionate)	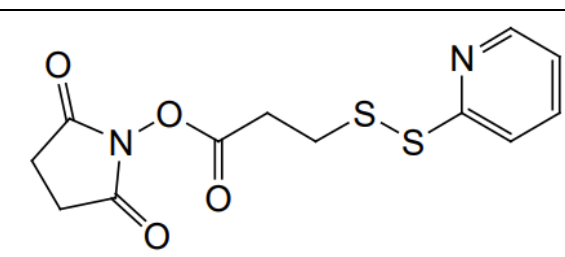	Bears a disulfide bond that can be cleaved by reduction	Crosslinking degree: 44%	We did not test this in cells because SPDP also bears a disulfide bond, like DSP, and we found that the cleavage efficiency of DSP was not high enough to give us a crack-free gel.

ThermoFisher	20665	DTBP (Dimethyl 3,3'-dithiobispropionimidate•2HCl)	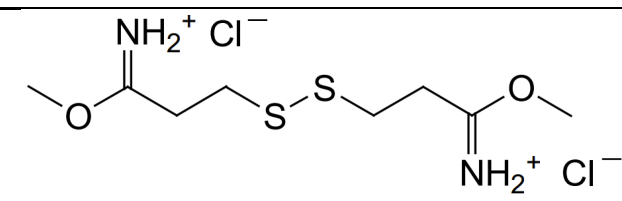	Bears a disulfide bond that can be cleaved by reduction	Crosslinking degree: 73%<br>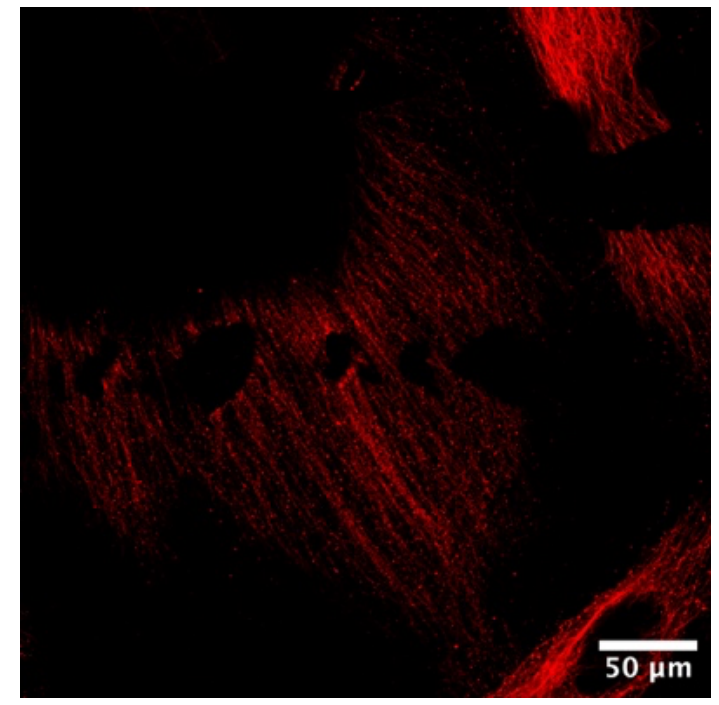<br>1.25 mM DTBP + 3% PFA, in PBS for 8h at RT, post-expansion stained against beta tubulin. The confocal image was acquired using a 40× water-immersion objective.	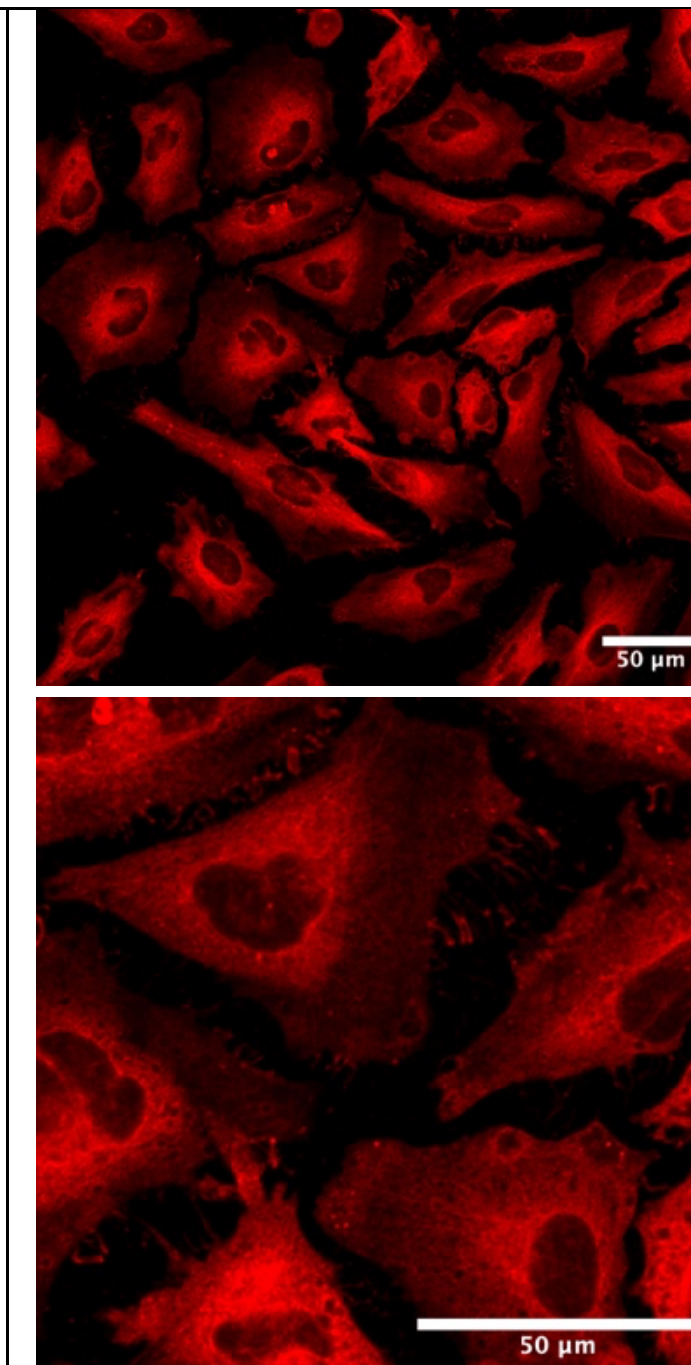<br>HeLa cells were fixed with 20mM DTBP in phosphate buffer (pH 8) incubated for 1h (without PFA, without ExM). The confocal image was acquired using a 40× water-immersion objective.<br><br>Tubulins were dispersed

ThermoFisher	21666	DMP (Dimethyl pimelimidate •2 HCl)	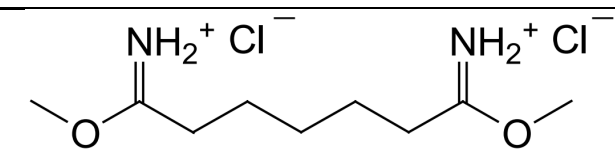	Bis(imidoester) crosslinkers form amidine bonds with primary amines, which have been reported to undergo hydrolysis at high pH, rendering the crosslinks reversible at high pH . In this study, we used DMA, DMP and DMS for in vitro crosslinking and did not test any high-pH cleavage protocol.	Crosslinking degree: 39%	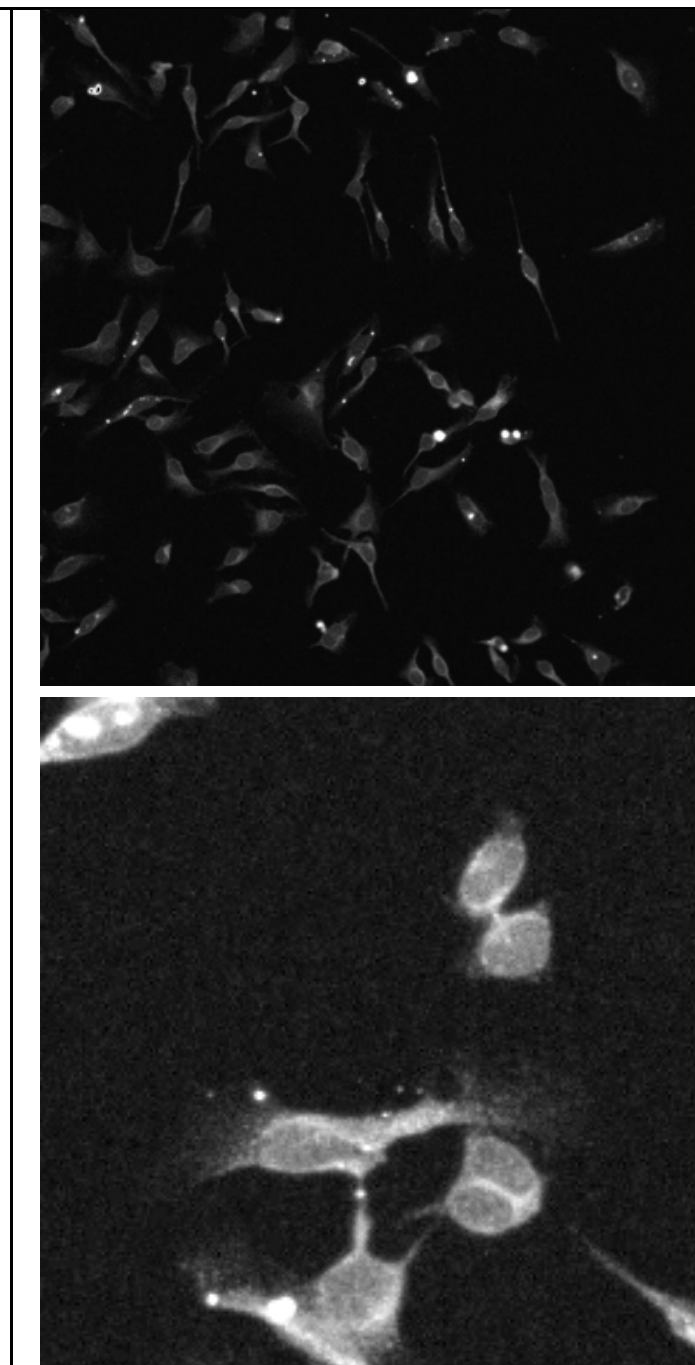 HeLa cells were fixed with 6.25 mM DMP + 1% PFA, in PBS, incubated for 6h at RT (fixation only, no ExM), and stained against beta tubulin. The confocal image was acquired using a 10× air objective.<br><br>Tubulins were dispersed.

						Raw confocal files were unavailable; therefore, scale bars are not shown.
ThermoFisher	20660	DMA (Dimethyl adipimidate•2 HCl)	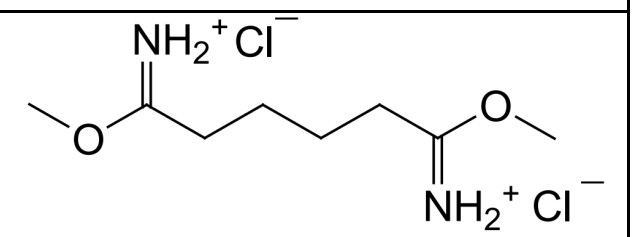		Crosslinking degree: 26%	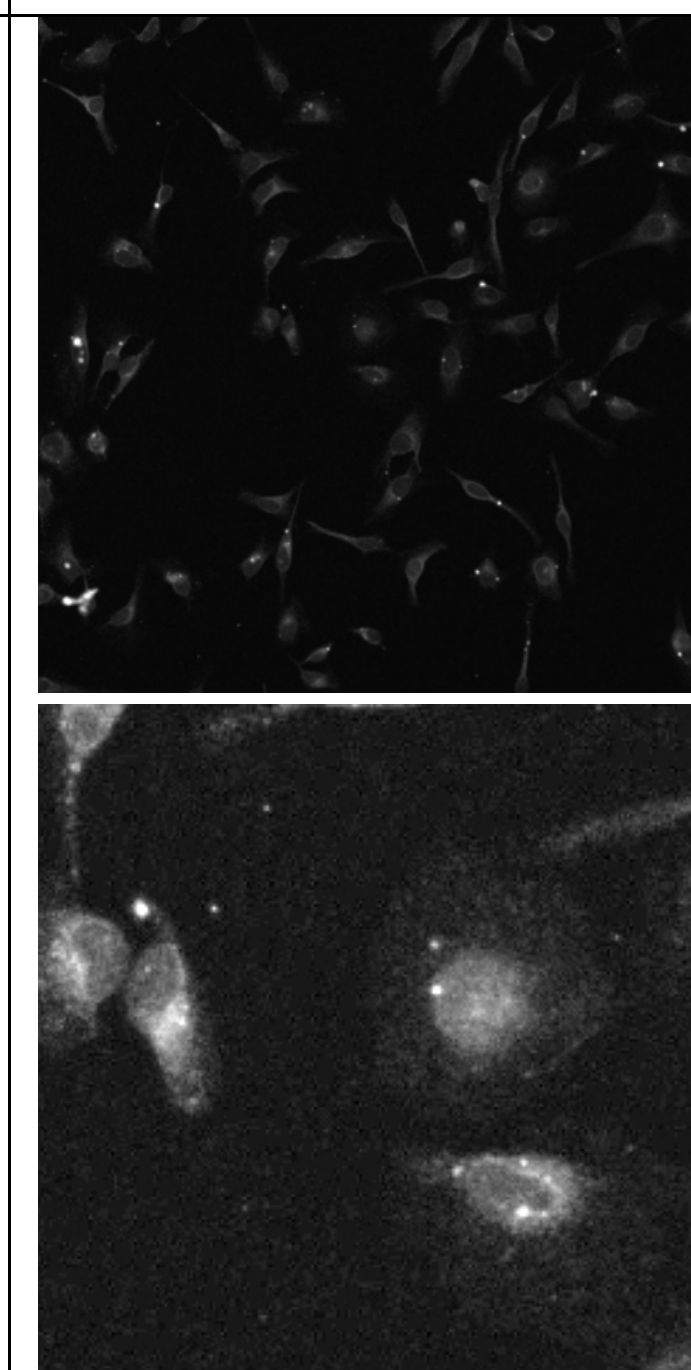 HeLa cells were fixed with 6.25 mM DMA + 1% PFA, in PBS, incubated for 6h at RT (fixation only, no ExM), and stained against beta tubulin. The confocal image was

						acquired using a 10× air objective.<br><br>Tubulins were dispersed.<br><br>Raw confocal files were unavailable; therefore, scale bars are not shown.
ThermoFisher	20700	DMS (Dimethyl suberimidate •2 HCl)	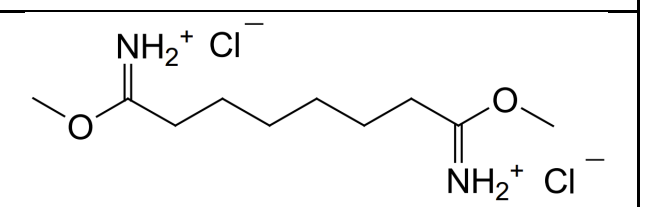		Crosslinking degree: 37%	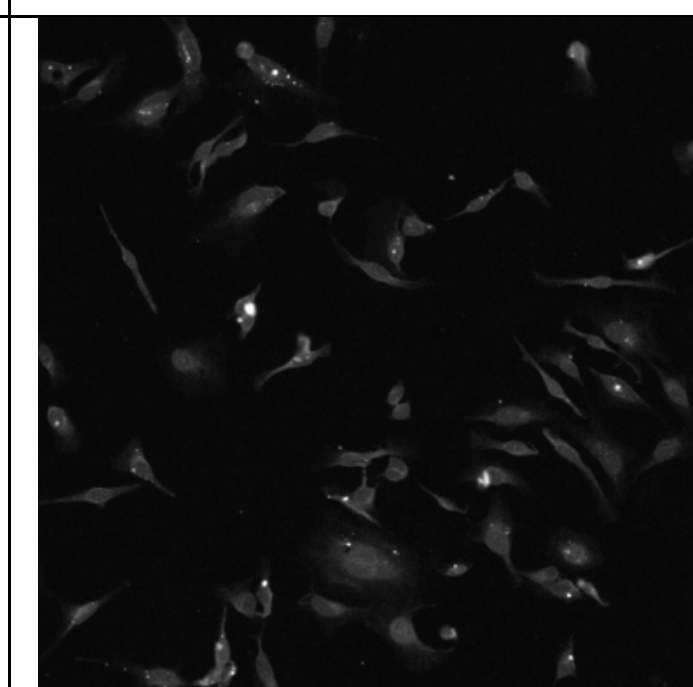<br>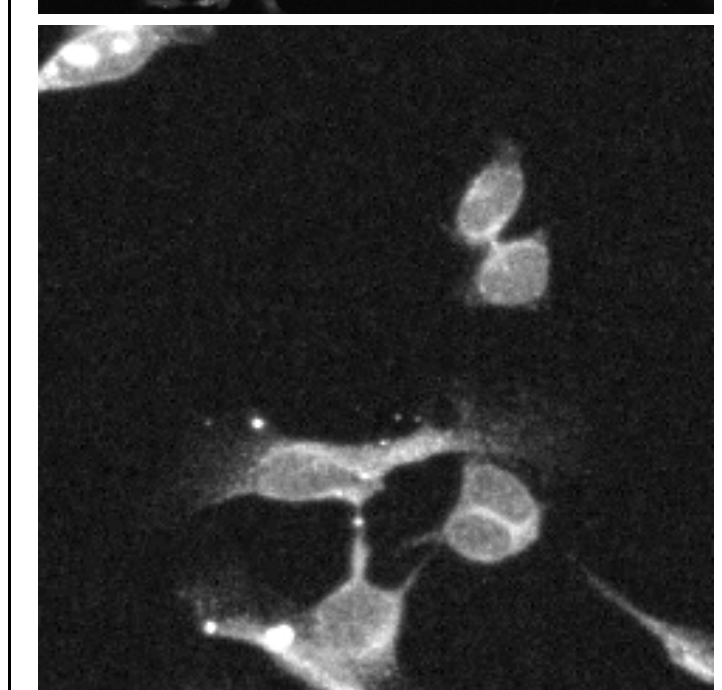<br>HeLa cells were fixed with 6.25 mM DMS + 1% PFA, in PBS, incubated

						for 6h at RT (fixation only, no ExM), and stained against beta tubulin. The confocal image was acquired using a 10× air objective.<br><br>Tubulins were dispersed<br><br>Raw confocal files were unavailable; therefore, scale bars are not shown.
ThermoFisher	21525	DFDNB (1,5-difluoro-2,4-dinitrobenzene)	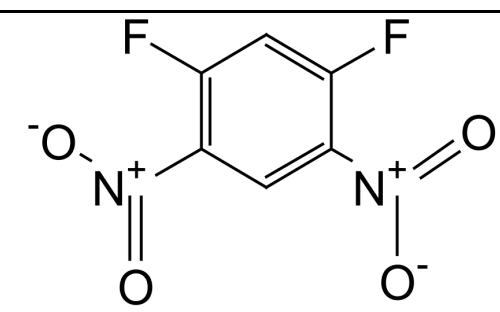	DFDNB contains aryl halide groups that can form cross-links with protein sulfhydryl groups and have been reported to be reversible upon treatment with excess thiol (e.g., DTT) (manufacture	No crosslinking (PAGE gel result)	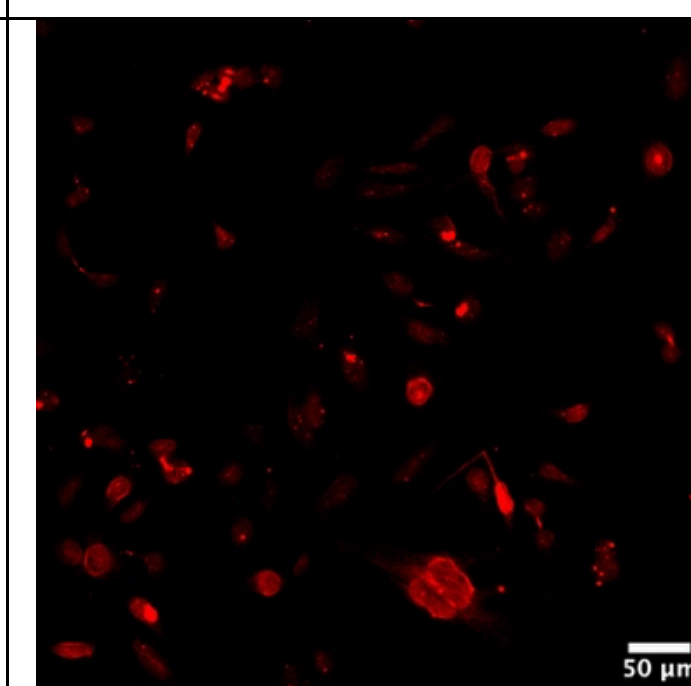

				r's instructions and ref. [15]). In this study, we only used DFDNB for crosslinking and did not test the cleavage step.		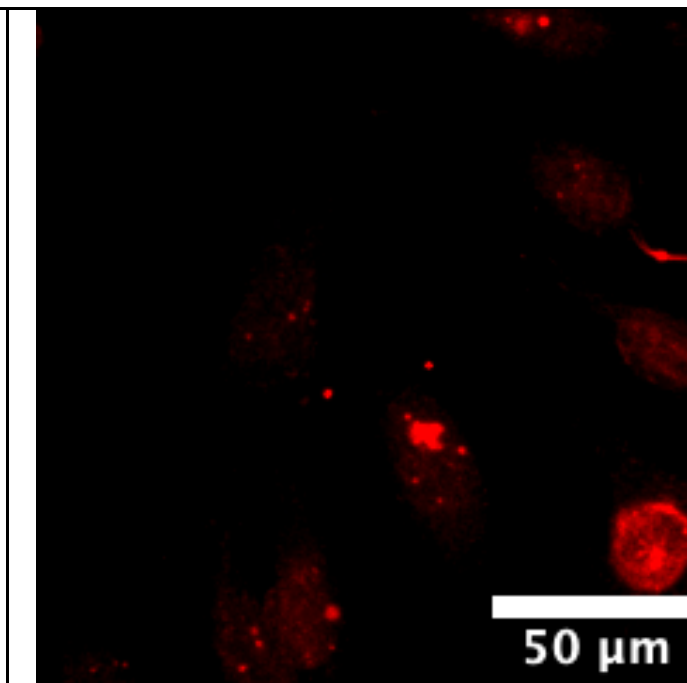<br>HeLa cells were fixed with 6.25 mM DFDNB + 1% PFA in PBS, incubated for 6h at RT (fixation only, no ExM), and stained against beta tubulin. The confocal image was acquired using a 10× air objective.<br><br>The cytoskeletal structure was almost completely gone.

Supplementary Table 3: Detailed Statistics for Figure 2

**Metric**	**Method**	**n**	**Mean ± SD**	**Median (IQR)**	**95% CI**
Mean Intensity (a.u.)	proExM(PFA)	75	42.74 ± 11.85	43.71 (33.12–50.59)	(40.01, 45.46)
Mean Intensity (a.u.)	CryoExM	124	76.24 ± 41.67	68.42 (49.71–98.81)	(68.83, 83.64)
Mean Intensity (a.u.)	nfExM	155	291.48 ± 88.03	284.60 (228.75–338.83)	(277.51, 305.45)
Gap Ratio (%)	proExM(PFA)	75	28.27 ± 5.20	28.57 (24.64–31.93)	(27.07, 29.46)
Gap Ratio (%)	CryoExM	124	24.17 ± 10.06	24.29 (16.67–30.16)	(22.38, 25.96)
Gap Ratio (%)	nfExM	155	1.80 ± 2.07	1.30 (0.00–2.93)	(1.48, 2.13)
Gap Size (µm)	proExM(PFA)	75	0.09 ± 0.02	0.09 (0.08–0.10)	(0.09, 0.10)
Gap Size (µm)	CryoExM	124	0.09 ± 0.02	0.09 (0.07–0.10)	(0.09, 0.09)
Gap Size (µm)	nfExM	155	0.03 ± 0.03	0.04 (0.00–0.04)	(0.03, 0.04)

Statistical comparisons (Mann–Whitney U test):

Metric	Comparison	P value	Significance
Mean Intensity (a.u.)	proExM(PFA) vs CryoExM	5.84e-12	****
Mean Intensity (a.u.)	proExM(PFA) vs nfExM	1.08e-34	****
Mean Intensity (a.u.)	CryoExM vs nfExM	8.76e-46	****
Gap Ratio (%)	proExM(PFA) vs CryoExM	3.35e-04	****
Gap Ratio (%)	proExM(PFA) vs nfExM	2.92e-35	****
Gap Ratio (%)	CryoExM vs nfExM	1.74e-46	****
Gap Size (µm)	proExM(PFA) vs CryoExM	0.050	ns
Gap Size (µm)	proExM(PFA) vs nfExM	1.80e-31	****
Gap Size (µm)	CryoExM vs nfExM	1.02e-38	****

*Significance levels: n.s. not significant, * p<0.05, ** p<0.01, *** p<0.001, **** p<0.0001*

Supplementary Table 4: Detailed Statistics for Figure 3

**Method**	**Intensity (a.u., Mean ± SD)**	**Gap Ratio (%, Mean ± SD)**	**Gap Size (µm, Mean ± SD)**	**N segments**
high T% ProExM	378.99 ± 95.62	11.58 ± 5.85	0.074 ± 0.029	59
nfExM	912.38 ± 144.47	1.65 ± 2.28	0.029 ± 0.032	61

Statistical comparisons (Mann-Whitney U-test):

Comparison	Parameter	p-value	Significance
nfExM vs. high T% ProExM	Intensity	3.60×10^{-21}	****
high T% ProExM vs. nfExM	Gap Ratio	1.52×10^{-16}	****
high T% ProExM vs. nfExM	Gap Size	2.49×10^{-11}	****

*Significance levels: **** p<0.0001*

Supplementary Table 5: Detailed Statistics for Figure 4

Method	Intensity (a.u., Mean ± SD)	Gap Ratio (%, Mean ± SD)	Gap Size (µm, Mean ± SD)	N segments
ProExM	503.64 ± 153.84	3.07 ± 3.55	0.038 ± 0.028	174
ExPath	502.38 ± 128.45	2.45 ± 3.01	0.037 ± 0.026	193
MAP	623.35 ± 201.87	2.75 ± 2.95	0.038 ± 0.032	145
nfExM	1029.47 ± 200.56	1.26 ± 1.88	0.029 ± 0.032	190

Statistical comparisons (Mann-Whitney U-test):

Comparison	Parameter	p-value	Significance
nfExM vs. ProExM	Intensity	5.68×10^{-55}	****
nfExM vs. ExPath	Intensity	2.08×10^{-60}	****
nfExM vs. MAP	Intensity	1.68×10^{-38}	****
ProExM vs. ExPath	Intensity	1.000	n.s.
ProExM vs. MAP	Intensity	3.04×10^{-7}	****
ExPath vs. MAP	Intensity	1.88×10^{-7}	****
ProExM vs. nfExM	Gap Ratio	1.57×10^{-7}	****
ExPath vs. nfExM	Gap Ratio	1.18×10^{-5}	****
MAP vs. nfExM	Gap Ratio	9.15×10^{-7}	****
ProExM vs. ExPath	Gap Ratio	1.000	n.s.
ProExM vs. MAP	Gap Ratio	1.000	n.s.
ExPath vs. MAP	Gap Ratio	1.000	n.s.
ProExM vs. nfExM	Gap Size	9.80×10^{-4}	***
ExPath vs. nfExM	Gap Size	0.004	**
MAP vs. nfExM	Gap Size	0.017	*
ProExM vs. ExPath	Gap Size	1.000	n.s.
ProExM vs. MAP	Gap Size	1.000	n.s.
ExPath vs. MAP	Gap Size	1.000	n.s.

P-values are Bonferroni-corrected for 6 pairwise comparisons (4 methods)

*Significance levels: n.s. not significant, * p<0.05, ** p<0.01, *** p<0.001, **** p<0.0001.*

Supplementary Table 6: Detailed Statistics for Figure 5

Metric	Method	n	Mean ± SD	Median (IQR)	95% CI

GM130 Signal Pixel Counts	CryoExM	16	42401.3 ± 29266.6	31563.5 (25747.8–52116.0)	(26806.3, 57996.4)
GM130 Mean Intensity (a.u.)	CryoExM	16	135.7 ± 19.4	137.9 (123.3–146.3)	(125.4, 146.0)
GM130 Signal Pixel Counts	nfExM	16	1355002.3 ± 457558.6	1322288.0 (1182253.5–1500040.0)	(1111186.5, 1598818.1)
GM130 Mean Intensity (a.u.)	nfExM	16	256.7 ± 32.1	255.9 (232.7–272.9)	(239.6, 273.8)
TOM20 Signal Pixel Counts	CryoExM	8	652449.8 ± 144015.6	640159.5 (559697.5–770053.0)	(532049.7, 772849.8)
TOM20 Mean Intensity (a.u.)	CryoExM	8	269.7 ± 47.3	258.1 (236.0–280.8)	(230.1, 309.3)
TOM20 Signal Pixel Counts	nfExM	8	1117785.2 ± 289200.7	1099425.0 (959146.8–1208696.5)	(876007.4, 1359563.1)
TOM20 Mean Intensity (a.u.)	nfExM	8	421.7 ± 48.9	414.9 (383.5–437.0)	(380.8, 462.6)

Statistical comparisons (Unpaired t-test):

Metric	Comparison	P value	Significance
GM130 Signal Pixel Counts	nfExM vs CryoExM	1.54e-06	****
GM130 Mean Intensity (a.u.)	nfExM vs CryoExM	1.54e-06	****
TOM20 Signal Pixel Counts	nfExM vs CryoExM	0.0011	***
TOM20 Mean Intensity (a.u.)	nfExM vs CryoExM	1.55e-04	****

*Significance levels: n.s. not significant, * p<0.05, ** p<0.01, *** p<0.001, **** p<0.0001.*

References

1. Yu, C.-C. (Jay) *et al.* Expansion microscopy of C. elegans. *Elife* **9**, e46249 (2020).
2. Simpson, R. J. Fragmentation of protein using trypsin. *Cold Spring Harb. Protoc.* **2006**, pdb.prot4550 (2006).
3. Cal, P. M. S. D. *et al.* Iminoboronates: a new strategy for reversible protein modification. *J. Am. Chem. Soc.* **134**, 10299–10305 (2012).
4. Bandyopadhyay, A. & Gao, J. Iminoboronate-based peptide cyclization that responds to pH, oxidation, and small molecule modulators. *J. Am. Chem. Soc.* **138**, 2098–2101 (2016).
5. Milo, R. & Phillips, R. *Cell Biology by the Numbers*. (Garland Science, Taylor & Francis Group, New York, NY, 2016).
6. Moran, U., Phillips, R. & Milo, R. SnapShot: key numbers in biology. *Cell* **141**, 1262-1262.e1 (2010).
7. Zarling, D. A., Watson, A. & Bach, F. H. Mapping of lymphocyte surface polypeptide antigens by chemical cross-linking with BSOCOES. *J. Immunol.* **124**, 913–920 (1980).
8. Sun, Y., Frenkel-Pinter, M., Liotta, C. L. & Grover, M. A. The pH dependent mechanisms of non-enzymatic peptide bond cleavage reactions. *Phys. Chem. Chem. Phys.* **22**, 107–113 (2019).
9. Tanaka, T., Inoue, M., Sakamoto, J. & Sone, N. Intra- and inter-complex cross-linking of subunits in the quinol oxidase super-complex from thermophilic bacillus PS31. *J. Biochem. (Tokyo)* **119**, 482–486 (1996).
10. Hermanson, G. T. *Bioconjugate Techniques*. (Academic Press, an imprint of Elsevier, Amsterdam Boston Heidelberg, 2013).
11. Trobo-Maseda, L., Orrego, A. H., Romero-Fernández, M., Guisan, J. M. & Rocha-Martín, J. Immobilization of enzymes on hetero-functional supports: physical adsorption plus additional covalent immobilization. in *Immobilization of enzymes and*

cells (eds Guisan, J. M., Bolivar, J. M., López-Gallego, F. & Rocha-Martín, J.) vol. 2100 159–174 (Springer US, New York, NY, 2020).

12. Mateo, C. *et al.* Immobilization of enzymes on heterofunctional epoxy supports. *Nat. Protoc.* **2**, 1022–1033 (2007).
13. Mclean, I. W. & Nakane, P. K. Periodate-lysine-paraformaldehyde fixative a new fixative for immunoelectron microscopy. *J. Histochem. Cytochem.* **22**, 1077–1083 (1974).
14. Günay, K. A. *et al.* Photo-expansion microscopy enables super-resolution imaging of cells embedded in 3D hydrogels. *Nat. Mater.* **22**, 777–785 (2023).
15. Shaltiel, S. Thiolysis of some dinitrophenyl derivatives of amino acids. *Biochem. Biophys. Res. Commun.* **29**, 178–183 (1967).
